# Supplementary material for: Electronic metal–support interaction modulates single-atom platinum catalysis for hydrogen evolution reaction
Source: Nat Commun. 2021 May 21;12:3021. doi: 10.1038/s41467-021-23306-6 (PMC8140142; doi:10.1038/s41467-021-23306-6)
Supplement: Supplementary file 1 — Supplementary Information [file 41467_2021_23306_MOESM1_ESM.pdf]

# Supplementary Information

# Electronic metal–support interaction modulates single-atom platinum catalysis for hydrogen evolution reaction

Shi et al.

## Supplementary Methods

### Synthesis of chemically exfoliated transition metal dichalcogenides (ce-TMDs) nanosheets

Bulk TMDs ($MoS_2$, $WS_2$, $MoSe_2$, or $WSe_2$) powder (0.6 g) were first mixed with n-butyllithium in cyclohexane (2.0 M, 8 ml) at 70 °C in argon-atmosphere glove box for 72 h. The resulting suspension was centrifuged and washed with 30 ml of cyclohexane for four times. After lithium intercalation, the black compound was immediately transferred in water and then ultra-sonicated for 30 min. The exfoliated suspension was further dialyzed for 3 days (dialysis membrane purchased from BIOSHARP, 14000 D). The suspension was then centrifuged to remove the unexfoliated materials. Finally, *ca.* 100 mL of the ce-TMDs supernatant (ce-$MoS_2$, ce-$WS_2$, ce-$MoSe_2$, or ce-$WSe_2$) was obtained and freshly used.

### DFT calculations

First-principle calculations were performed by using the Vienna ab initio simulation package (VASP 5.4.4) with the Perdew–Burke–Ernzerhof generalized gradient approximation for the exchange-correlation functional and the projector augmented wave method. The DFT-D2 method was adopted to describe the van der Waals interactions between the adsorbed atoms and the support. A 2 × 3 × 1 supercell of $MX_2$ (X=S, Se; M=Mo, W) and a vacuum region of 18 Å were used to eliminate interactions between the neighboring cells of slab models for all calculations. The plane-wave cutoff energy was set as 400 eV for all calculations; 3 × 3 × 1 and 5 × 5 × 1 Γ-centered k points were adopted for geometry optimization and self-consistent electronic calculation, respectively. All the atoms were relaxed until the residual force was less than 0.01 eV/Å and the self-consistent field tolerance level was $1.0 \times 10^{-5}$ a.u. for the geometry optimizations.

The free energy of the adsorbed state was calculated as follows:

$$\Delta G_{H*} = \Delta E_{H*} + \Delta E_{ZPE} - T\Delta S \qquad \text{(S-1)}$$

where $\Delta E_{H*}$ is the energy of hydrogen adsorption, and $\Delta E_{ZPE}$ is the difference in zero-point energy between the adsorbed states and the gas phase. The hydrogen adsorption site was confirmed by comparing the energy of hydrogen adsorption at different positions on the catalyst surface after free geometry optimization with an optimization tolerance level of $2.0 \times 10^{-5}$ a.u.. The van der Waals correction was taken into consideration in all these calculations.

## Supplementary Notes

### Supplementary Note 1. Calculation of mass activity for HER

Alkaline HER ($\eta$@100 mV):

Pt-SAs/$MoS_2$:

$$\frac{24.38 \times 0.07065 \times 10^{-3}}{5 \times 10^{-3} \times 5.1\%} = 6.75\, A\, mg^{-1}$$

Pt-SAs/$MoSe_2$:

$$\frac{99.47 \times 0.07065 \times 10^{-3}}{4.35 \times 10^{-3} \times 4.7\%} = 34.37\, A\, mg^{-1}$$

Pt-SAs/$WS_2$:

$$\frac{8.01 \times 0.07065 \times 10^{-3}}{5.6 \times 10^{-3} \times 4.1\%} = 2.46\, A\, mg^{-1}$$

Pt-SAs/$WSe_2$:

$$\frac{7.63 \times 0.07065 \times 10^{-3}}{5 \times 10^{-3} \times 4.9\%} = 2.20\, A\, mg^{-1}$$

Commercial Pt/C:

$$\frac{13.25 \times 0.07065 \times 10^{-3}}{2 \times 5 \times 10^{-3} \times 20\%} = 0.468\, A\, mg^{-1}$$

Acidic HER ($\eta$@100 mV):

Pt-SAs/$MoS_2$:

$$\frac{21.42 \times 0.07065 \times 10^{-3}}{6.25 \times 10^{-4} \times 5.1\%} = 47.48\, A\, mg^{-1}$$

Pt-SAs/$MoSe_2$:

$$\frac{15.97 \times 0.07065 \times 10^{-3}}{5.5 \times 10^{-4} \times 4.7\%} = 43.65\ A\ mg^{-1}$$

Pt-SAs/$WS_2$:

$$\frac{54.03 \times 0.07065 \times 10^{-3}}{7.15 \times 10^{-4} \times 4.1\%} = 130.21\ A\ mg^{-1}$$

Pt-SAs/$WSe_2$:

$$\frac{5.05 \times 0.07065 \times 10^{-3}}{6.5 \times 10^{-4} \times 4.9\%} = 11.20\ A\ mg^{-1}$$

Commercial Pt/C:

$$\frac{85.6 \times 0.07065 \times 10^{-3}}{2 \times 5 \times 10^{-3} \times 20\%} = 3.02\ A\ mg^{-1}$$

**Supplementary Note 2. Calculation of TOFs for HER**

**Alkaline HER:**

Pt-SAs/$MoS_2$:

$$n = \frac{5.0 \times 10^{-3} \times 5.1\% \times 10^{-3}}{195} = 1.31 \times 10^{-9}\, mol$$

$$\mathrm{TOF(@0.05\ V)} = \frac{I}{2F \times n} = \frac{3.64 \times 0.07065 \times 10^{-3}}{2 \times 96500 \times 1.31 \times 10^{-9}} = 1.02\, s^{-1}$$

$$\mathrm{TOF(@0.10\ V)} = \frac{I}{2F \times n} = \frac{24.38 \times 0.07065 \times 10^{-3}}{2 \times 96500 \times 1.31 \times 10^{-9}} = 6.80\, s^{-1}$$

$$\mathrm{TOF(@0.15\ V)} = \frac{I}{2F \times n} = \frac{72.85 \times 0.07065 \times 10^{-3}}{2 \times 96500 \times 1.31 \times 10^{-9}} = 20.34\, s^{-1}$$

$$\mathrm{TOF(@0.20\ V)} = \frac{I}{2F \times n} = \frac{150.05 \times 0.07065 \times 10^{-3}}{2 \times 96500 \times 1.31 \times 10^{-9}} = 41.86\, s^{-1}$$

Pt-SAs/$MoSe_2$:

$$n = \frac{4.35 \times 10^{-3} \times 4.7\% \times 10^{-3}}{195} = 1.05 \times 10^{-9}\, mol$$

$$\mathrm{TOF(@0.05\ V)} = \frac{I}{2F \times n} = \frac{17.79 \times 0.07065 \times 10^{-3}}{2 \times 96500 \times 1.05 \times 10^{-9}} = 6.21\, s^{-1}$$

$$\mathrm{TOF(@0.10\ V)} = \frac{I}{2F \times n} = \frac{99.47 \times 0.07065 \times 10^{-3}}{2 \times 96500 \times 1.05 \times 10^{-9}} = 34.69\, s^{-1}$$

$$\mathrm{TOF(@0.15\ V)} = \frac{I}{2F \times n} = \frac{189.44 \times 0.07065 \times 10^{-3}}{2 \times 96500 \times 1.05 \times 10^{-9}} = 66.07\, s^{-1}$$

$$\mathrm{TOF(@0.20\ V)} = \frac{I}{2F \times n} = \frac{289.07 \times 0.07065 \times 10^{-3}}{2 \times 96500 \times 1.05 \times 10^{-9}} = 100.82\, s^{-1}$$

Pt-SAs/$WS_2$:

$$n = \frac{5.6 \times 10^{-3} \times 4.1\% \times 10^{-3}}{195} = 1.18 \times 10^{-9}\, mol$$

$$\text{TOF(@0.05 V)} = \frac{I}{2F \times n} = \frac{2.63 \times 0.07065 \times 10^{-3}}{2 \times 96500 \times 1.18 \times 10^{-9}} = 0.56\, s^{-1}$$

$$\text{TOF(@0.10 V)} = \frac{I}{2F \times n} = \frac{8.01 \times 0.07065 \times 10^{-3}}{2 \times 96500 \times 1.18 \times 10^{-9}} = 2.49\, s^{-1}$$

$$\text{TOF(@0.15 V)} = \frac{I}{2F \times n} = \frac{20.58 \times 0.07065 \times 10^{-3}}{2 \times 96500 \times 1.18 \times 10^{-9}} = 6.41\, s^{-1}$$

$$\text{TOF(@0.20 V)} = \frac{I}{2F \times n} = \frac{47.74 \times 0.07065 \times 10^{-3}}{2 \times 96500 \times 1.18 \times 10^{-9}} = 14.87\, s^{-1}$$

Pt-SAs/$WSe_2$:

$$n = \frac{5.0 \times 10^{-3} \times 4.9\% \times 10^{-3}}{195} = 1.26 \times 10^{-9}\, mol$$

$$\text{TOF(@0.05 V)} = \frac{I}{2F \times n} = \frac{1.80 \times 0.07065 \times 10^{-3}}{2 \times 96500 \times 1.26 \times 10^{-9}} = 0.52\, s^{-1}$$

$$\text{TOF(@0.10 V)} = \frac{I}{2F \times n} = \frac{7.63 \times 0.07065 \times 10^{-3}}{2 \times 96500 \times 1.26 \times 10^{-9}} = 2.20\, s^{-1}$$

$$\text{TOF(@0.15 V)} = \frac{I}{2F \times n} = \frac{16.27 \times 0.07065 \times 10^{-3}}{2 \times 96500 \times 1.26 \times 10^{-9}} = 4.70\, s^{-1}$$

$$\text{TOF(@0.20 V)} = \frac{I}{2F \times n} = \frac{28.43 \times 0.07065 \times 10^{-3}}{2 \times 96500 \times 1.26 \times 10^{-9}} = 8.21\, s^{-1}$$

**Acidic HER:**

Pt-SAs/$MoS_2$:

$$n = \frac{6.25 \times 10^{-4} \times 5.1\% \times 10^{-3}}{195} = 1.64 \times 10^{-10}\ \text{mol}$$

$$\text{TOF(@0.05 V)} = \frac{I}{2F \times n} = \frac{5.75 \times 0.07065 \times 10^{-3}}{2 \times 96500 \times 1.64 \times 10^{-10}} = 12.83\ \text{s}^{-1}$$

$$\text{TOF(@0.10 V)} = \frac{I}{2F \times n} = \frac{21.42 \times 0.07065 \times 10^{-3}}{2 \times 96500 \times 1.64 \times 10^{-10}} = 47.77\ \text{s}^{-1}$$

$$\text{TOF(@0.15 V)} = \frac{I}{2F \times n} = \frac{46.50 \times 0.07065 \times 10^{-3}}{2 \times 96500 \times 1.64 \times 10^{-10}} = 103.75\ \text{s}^{-1}$$

$$\text{TOF(@0.20 V)} = \frac{I}{2F \times n} = \frac{77.06 \times 0.07065 \times 10^{-3}}{2 \times 96500 \times 1.64 \times 10^{-10}} = 171.95\ \text{s}^{-1}$$

Pt-SAs/$WS_2$:

$$n = \frac{7.15 \times 10^{-4} \times 4.1\% \times 10^{-3}}{195} = 1.50 \times 10^{-10}\ \text{mol}$$

$$\text{TOF(@0.05 V)} = \frac{I}{2F \times n} = \frac{20.61 \times 0.07065 \times 10^{-3}}{2 \times 96500 \times 1.50 \times 10^{-10}} = 50.22\ \text{s}^{-1}$$

$$\text{TOF(@0.10 V)} = \frac{I}{2F \times n} = \frac{54.03 \times 0.07065 \times 10^{-3}}{2 \times 96500 \times 1.50 \times 10^{-10}} = 131.65\ \text{s}^{-1}$$

$$\text{TOF(@0.15 V)} = \frac{I}{2F \times n} = \frac{83.46 \times 0.07065 \times 10^{-3}}{2 \times 96500 \times 1.50 \times 10^{-10}} = 203.36\ \text{s}^{-1}$$

$$\text{TOF(@0.20 V)} = \frac{I}{2F \times n} = \frac{111.91 \times 0.07065 \times 10^{-3}}{2 \times 96500 \times 1.50 \times 10^{-10}} = 272.68\ \text{s}^{-1}$$

Pt-SAs/$MoSe_2$:

$$n = \frac{5.5 \times 10^{-4} \times 4.7\% \times 10^{-3}}{195} = 1.33 \times 10^{-10}\ \text{mol}$$

$$\text{TOF(@0.05 V)} = \frac{I}{2F \times n} = \frac{4.37 \times 0.07065 \times 10^{-3}}{2 \times 96500 \times 1.33 \times 10^{-10}} = 12.03\ \text{s}^{-1}$$

$$\mathrm{TOF}(@0.10\ \mathrm{V}) = \frac{I}{2F \times n} = \frac{15.97 \times 0.07065 \times 10^{-3}}{2 \times 96500 \times 1.33 \times 10^{-10}} = 43.98\ \mathrm{s}^{-1}$$

$$\mathrm{TOF}(@0.15\ \mathrm{V}) = \frac{I}{2F \times n} = \frac{31.36 \times 0.07065 \times 10^{-3}}{2 \times 96500 \times 1.33 \times 10^{-10}} = 86.36\ \mathrm{s}^{-1}$$

$$\mathrm{TOF}(@0.20\ \mathrm{V}) = \frac{I}{2F \times n} = \frac{54.71 \times 0.07065 \times 10^{-3}}{2 \times 96500 \times 1.33 \times 10^{-10}} = 150.65\ \mathrm{s}^{-1}$$

Pt-SAs/$WSe_2$:

$$n = \frac{6.5 \times 10^{-4} \times 4.9\% \times 10^{-3}}{195} = 1.63 \times 10^{-10}\ \mathrm{mol}$$

$$\mathrm{TOF}(@0.05\ \mathrm{V}) = \frac{I}{2F \times n} = \frac{1.3 \times 0.07065 \times 10^{-3}}{2 \times 96500 \times 1.63 \times 10^{-10}} = 2.92\ \mathrm{s}^{-1}$$

$$\mathrm{TOF}(@0.10\ \mathrm{V}) = \frac{I}{2F \times n} = \frac{5.05 \times 0.07065 \times 10^{-3}}{2 \times 96500 \times 1.63 \times 10^{-10}} = 11.34\ \mathrm{s}^{-1}$$

$$\mathrm{TOF}(@0.15\ \mathrm{V}) = \frac{I}{2F \times n} = \frac{19.34 \times 0.07065 \times 10^{-3}}{2 \times 96500 \times 1.63 \times 10^{-10}} = 43.43\ \mathrm{s}^{-1}$$

$$\mathrm{TOF}(@0.20\ \mathrm{V}) = \frac{I}{2F \times n} = \frac{44.87 \times 0.07065 \times 10^{-3}}{2 \times 96500 \times 1.63 \times 10^{-10}} = 100.77\ \mathrm{s}^{-1}$$

**Supplementary Note 3. Comparison of single-atom Pt adsorbed atop $MoS_2$ and Pt-doped $MoS_2$ cases**

In order to elucidate the advantage of single-atom Pt supported atop TMDs (Pt-SAs/TMDs in this work) for the atomic-level electronic modulation for HER enhancement, we further compared the adsorbed single-atom Pt with the previously reported Pt-doped $MoS_2$ material[1]. In our system, we can see that single-atom Pt adsorbed atop $MoS_2$ (Pt-SAs/$MoS_2$) was dramatically poisoned by $SCN^-$ owing to the blocking of active Pt sites, resulting in a large decrease of HER current approaching zero (Supplementary Fig. 26). Similarly, Bao et al. also found that the activity of Pt adsorbed on $MoS_2$ drops quickly after adding methanol because the exposed Pt atoms atop $MoS_2$ support were easily poisoned by methanol[1]. On the contrary, Pt-doped $MoS_2$ showed negligible current decrease and excellent poisoning resistance ability with the introduction of methanol[1]. Through DFT calculations, owing to the steric hindrance, the H atom cannot adsorb on the doped Pt atom, while the preferred adsorption site is the neighboring unsaturated S atom with $\Delta G_H$ of ~0 eV (Supplementary Tab. 8). In the adsorbed Pt case, the preferred adsorption site for H atom is the Pt atom with $\Delta G_H$ of also ~0 eV, while the neighboring S atom shows very large $\Delta G_H$ value of ~1.94 eV (Supplementary Tab. 8). In this case, they came to the conclusion that the active sites of HER in such Pt-doped $MoS_2$ should originate from the S atoms rather than the Pt atoms. Compared with the Pt-doping case, the single-atom Pt adsorbed atop $MoS_2$ in our work can expose more unsaturated active sites to reactive species, which reduces the influence of ce-TMDs support for HER enhancement.

Additionally, unlike 2H-$MoS_2$ where the catalytic activity arises from the edges, experimental and theoretical results from Chhowalla and Jiang et al. suggested that the active sites of the chemically exfoliated 1T $MoS_2$ nanosheets are mainly the S atoms located on the basal plane and the contribution of the metallic edges to the overall HER efficiency is relatively small[2,3]. Thus, the Pt attachment on chalcogen atoms in the basal plane of 1T-TMDs can largely reduce the influence of TMDs supports for electrocatalytic HER, owing to the occupation of active sites of 1T-TMDs and the steric hindrance.

## Supplementary Figures

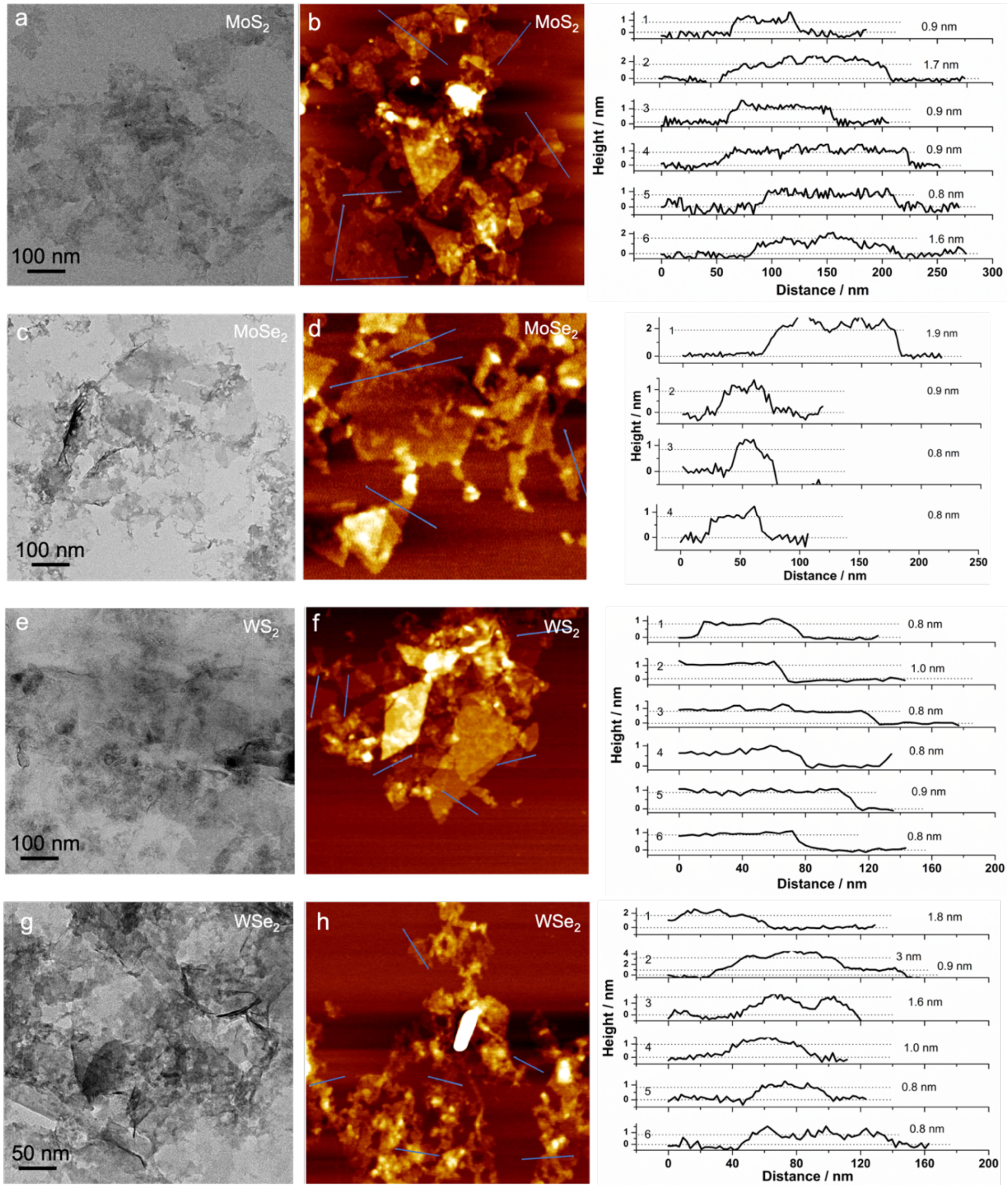


**Supplementary Figure 1.** (a, c, e, g) TEM images of the freshly prepared ce-TMDs nanosheets as indicated. (b, d, f, h) AFM analyses for the freshly prepared ce-TMDs materials as indicated and their corresponding height values.

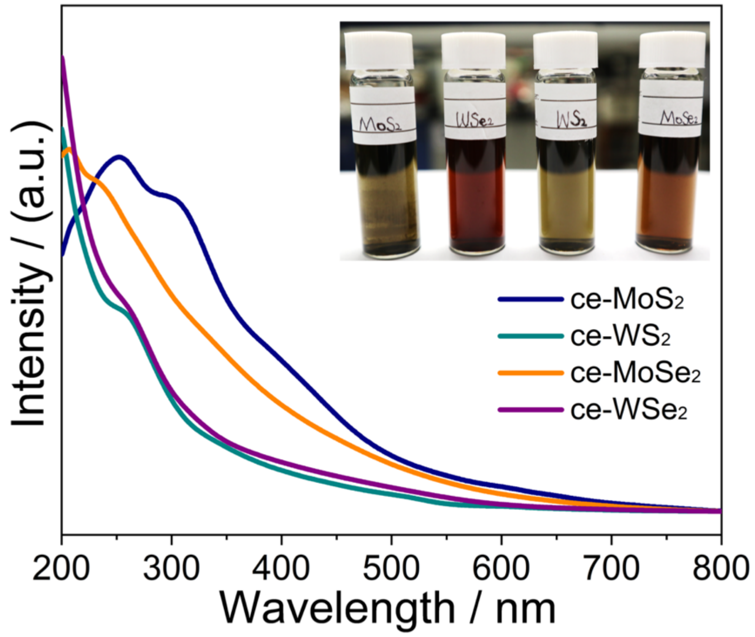


**Supplementary Figure 2.** UV-visible spectra of ce-TMDs suspensions. UV-visible spectra have been widely used as a facile structural characterization for ce-TMDs suspensions. The aqueous dispersion of all ce-TMDs samples showed no obvious characteristic peaks between 500~800 nm deriving from the optical band gap absorption of 2H phase, which indicated the dominant 1T phase of all the exfoliated TMDs samples. Inset shows the photograph of good-dispersible ce-TMDs nanosheets suspension in water. As observed, the water-dispersible ce-TMDs suspensions after diluting maintained excellent colloidal stability.

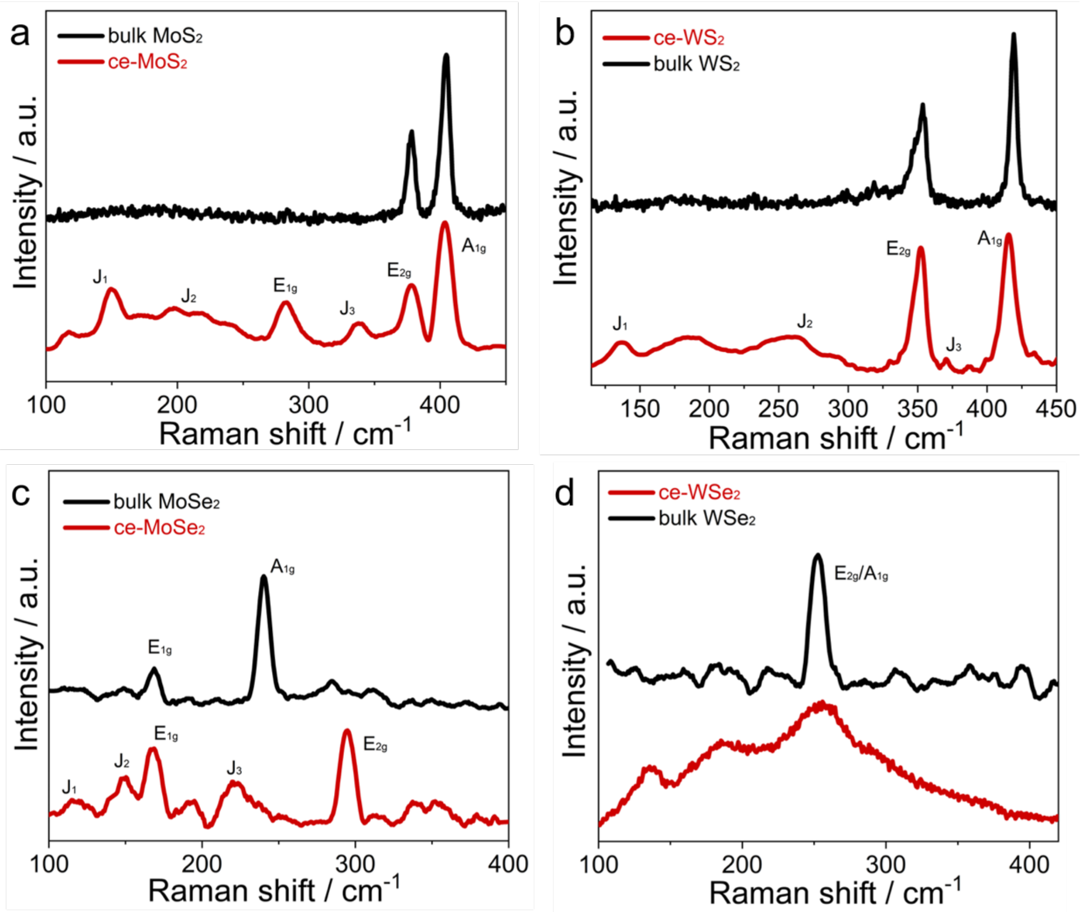


**Supplementary Figure 3.** Raman spectra of the ce-$MoS_2$ (a), ce-$WS_2$ (b), ce-$MoSe_2$ (c), and ce-$WSe_2$ (d) nanosheets (red lines), and its corresponding bulk TMDs samples (black lines). For the 2H-phase bulk TMDs samples, the characteristic peaks (*e.g.* $E_{2g}$ and $A_{1g}$) were very sharp. After chemical exfoliation, they became relatively much broader, and some new peak (*e.g.* $J_1$, $J_2$, or $J_3$) in the lower Raman shift regions appeared, indicating the structure transformation from 2H to 1T[4-8]. For example, apart from the $E_{2g}$ and $A_{1g}$ peaks of 2H-$MoS_2$, the new peaks at *ca.* 150, 221, and 335 $cm^{-1}$, ascribed to the $J_1$, $J_2$, and $J_3$ characteristic peaks of 1T-$MoS_2$, were also observed.

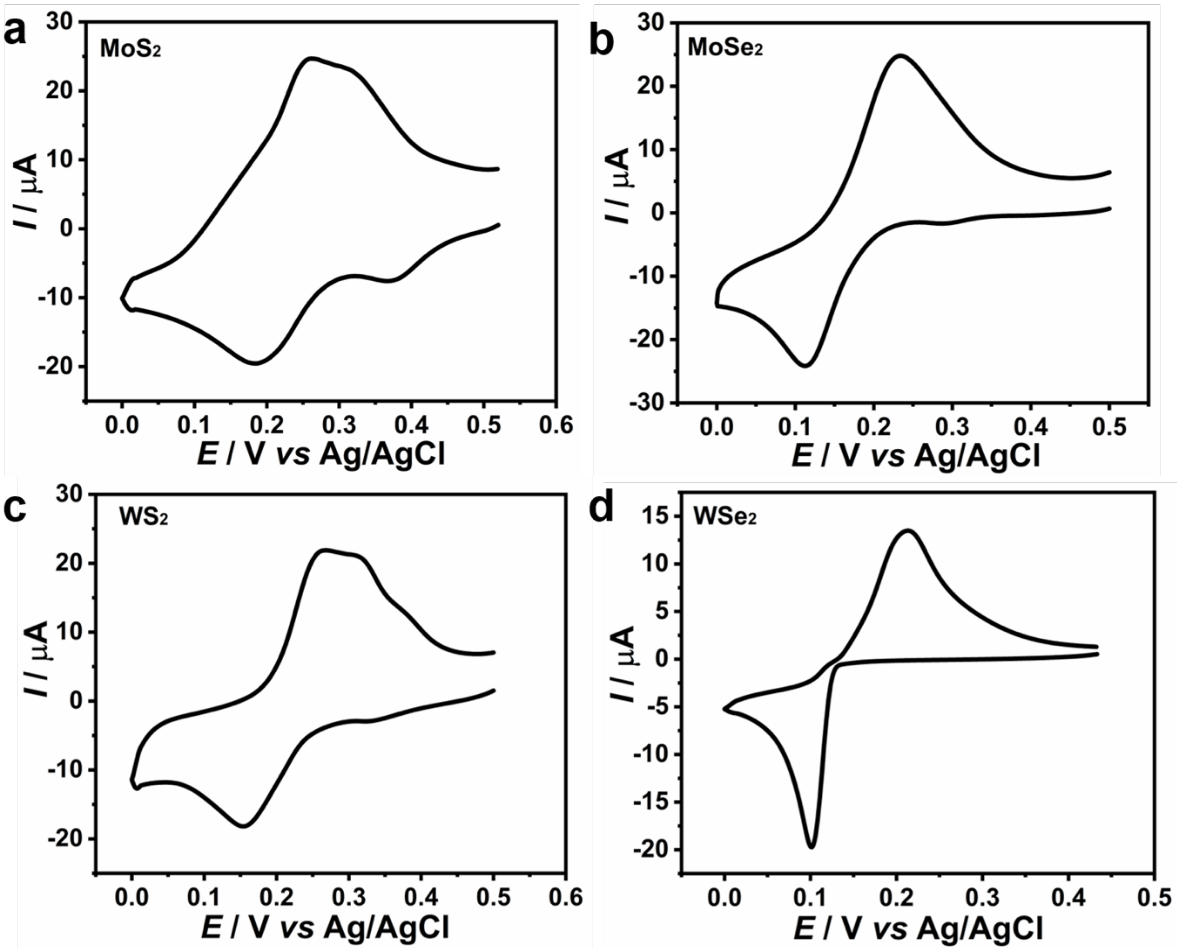


**Supplementary Figure 4.** Cyclic voltammograms (CVs) of a ce-$MoS_2$ (a), ce-$MoSe_2$ (b), ce-$WS_2$ (c), and ce-$WSe_2$ (d) modified GCE in an Ar-saturated 0.1 M $H_2SO_4$ + 2 mM $CuSO_4$ solution at a scan rate of 50 mV $s^{-1}$. The notable cathodic peaks in the CVs corresponded to the underpotential deposition of Cu on the ce-TMDs, while the anodic peaks in the opposite potential scan were attributed to the dissolution of Cu adatoms.

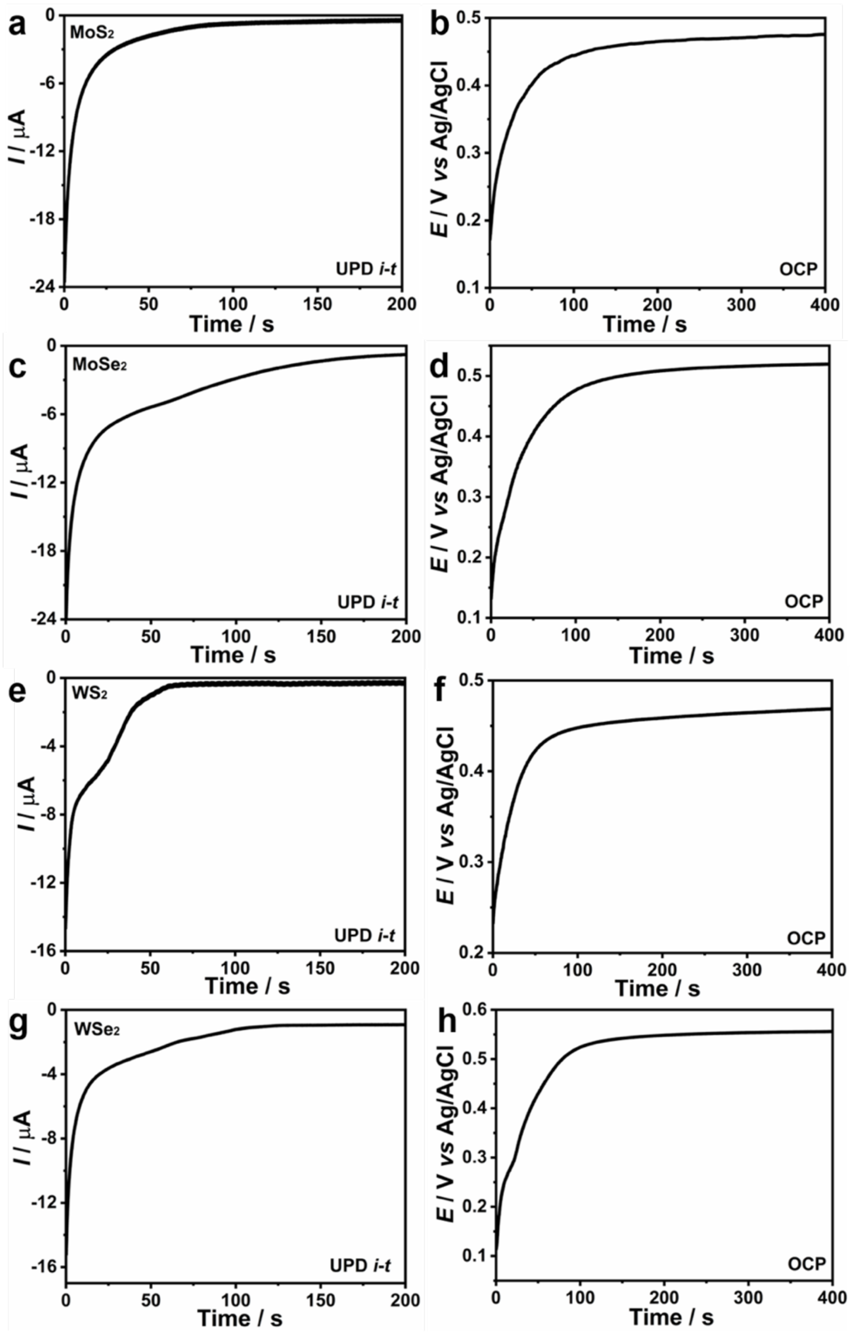


**Supplementary Figure 5.** Chronoamperometry curve of UPD of Cu on ce-$MoS_2$ (a), ce-$MoSe_2$ (c), ce-$WS_2$ (e), and ce-$WSe_2$ (g) nanosheets. Electroless galvanic replacement of Cu adatoms on ce-$MoS_2$ (b), ce-$MoSe_2$ (d), ce-$WS_2$ (f), and ce-$WSe_2$ (h) with $Pt^{II}$ at the open-circuit potential (OCP). The potential reached a plateau after *ca.* 150 s, suggesting a complete galvanic replacement of Cu with Pt.

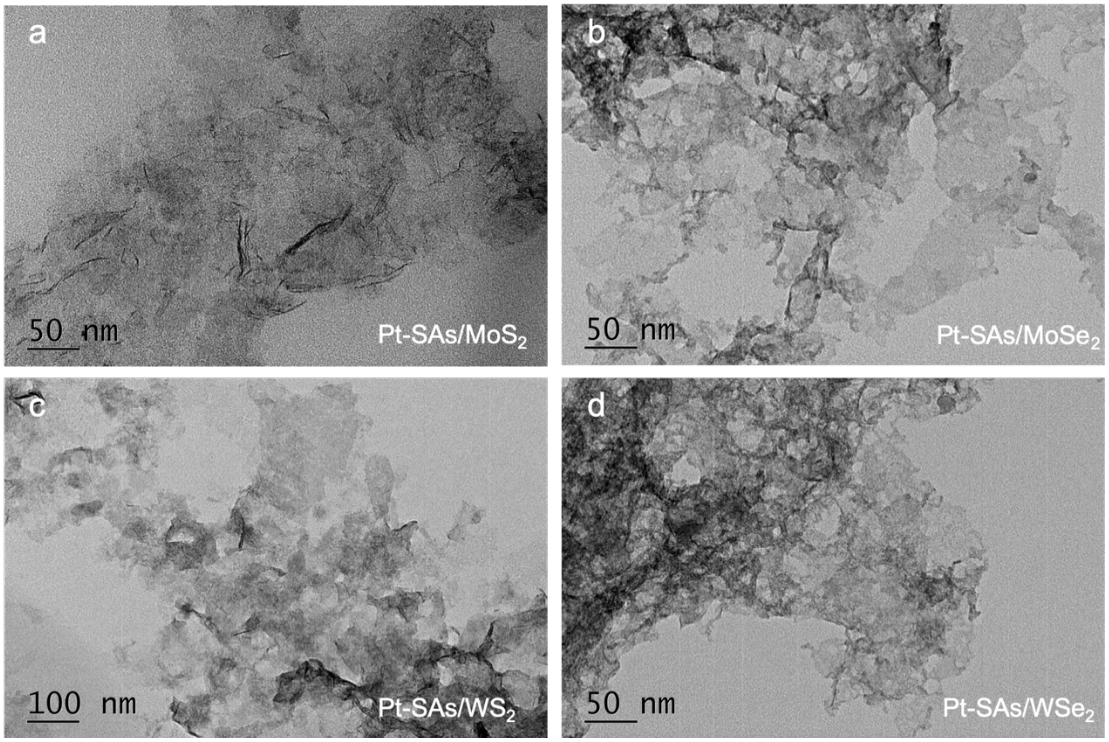


**Supplementary Figure 6.** Conventional TEM images of the freshly prepared Pt-SAs/$MoS_2$ (a), Pt-SAs/$MoSe_2$ (b), Pt-SAs/$WS_2$ (c), and Pt-SAs/$WSe_2$ (d) samples. After the decoration of Pt single atoms, the morphology of ce-TMDs remained sheet-like. Note that no obvious clusters or nanoparticles were found in Pt-SAs/TMDs, implying that most of the Pt existed in the atomically dispersed form.

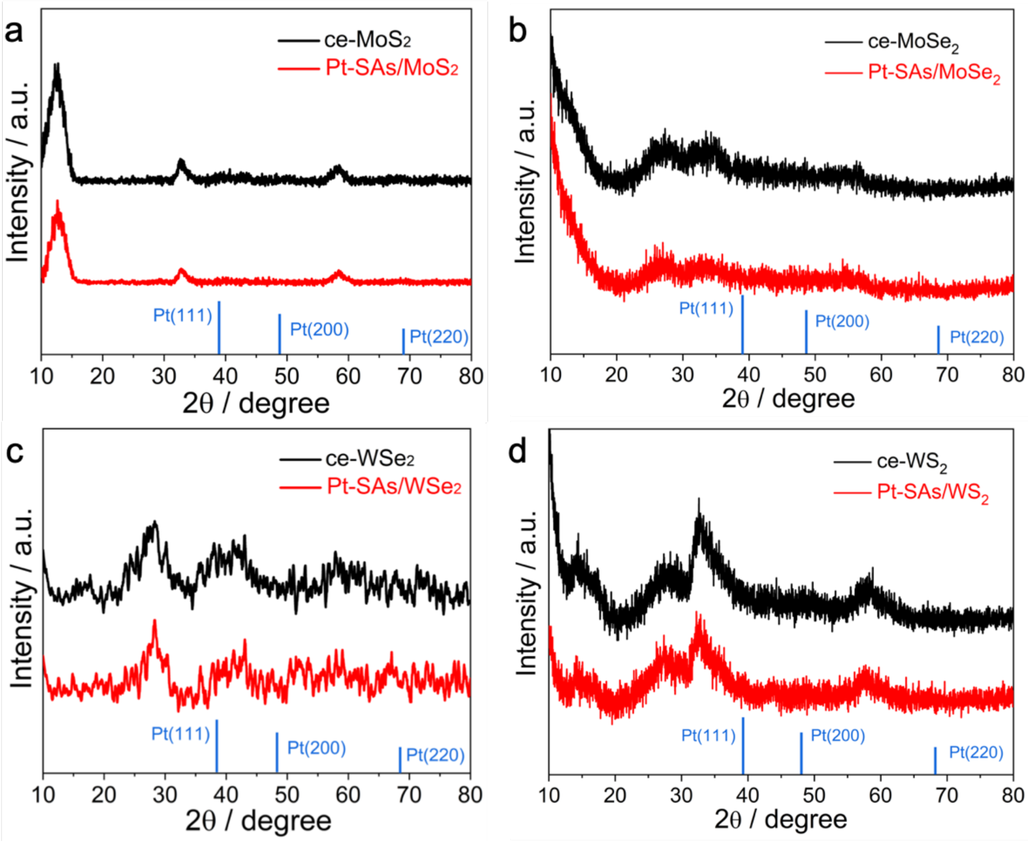


**Supplementary Figure 7.** XRD patterns of the Pt-SAs/$MoS_2$ (a), Pt-SAs/$MoSe_2$ (b), Pt-SAs/$WSe_2$ (c), and Pt-SAs/$WS_2$ (d) samples (red lines), and their corresponding pure ce-TMDs samples (black lines). For example, the diffraction peaks at 13.9°, 32.92°, 39.10°, and 58.42° are assigned to the (002), (100), (103), and (110) planes of $MoS_2$, respectively. No characteristic Pt peaks (JCPDS no.: 04-0802 Pt) were observed for all Pt-SAs/TMDs samples, indicative of the successful formation of atomically dispersed Pt.

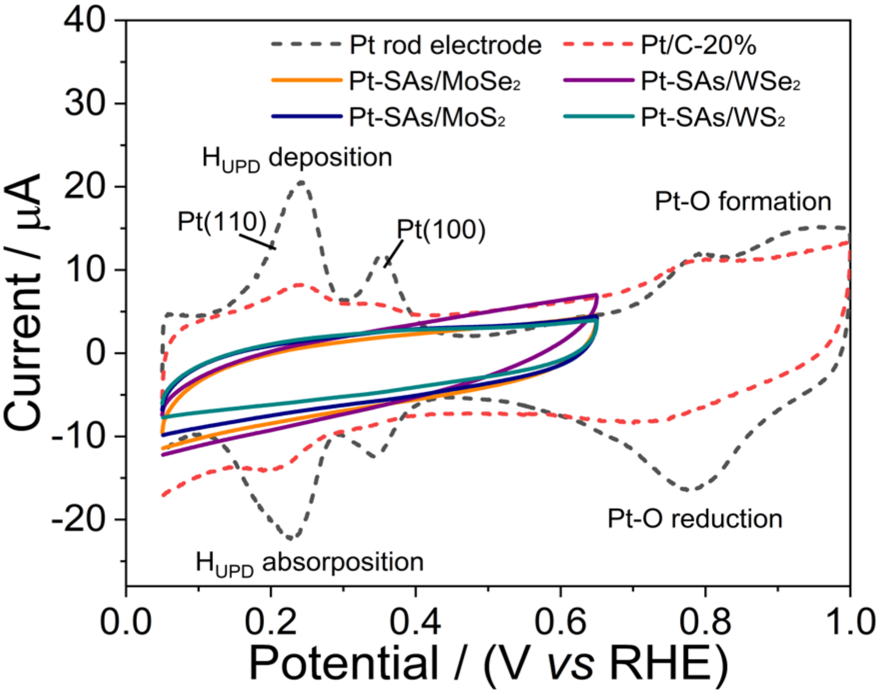


**Supplementary Figure 8.** CVs for various Pt-based materials in Ar-saturated 1.0 M KOH solution at a scan rate of 50 mV $s^{-1}$.

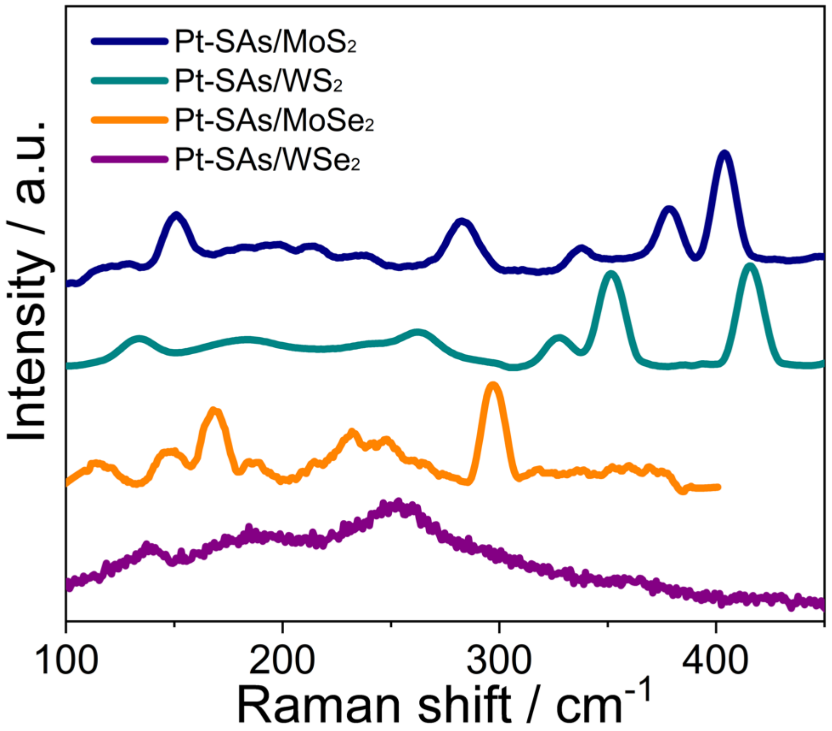


**Supplementary Figure 9.** Raman spectra of the Pt-SAs/TMDs samples.

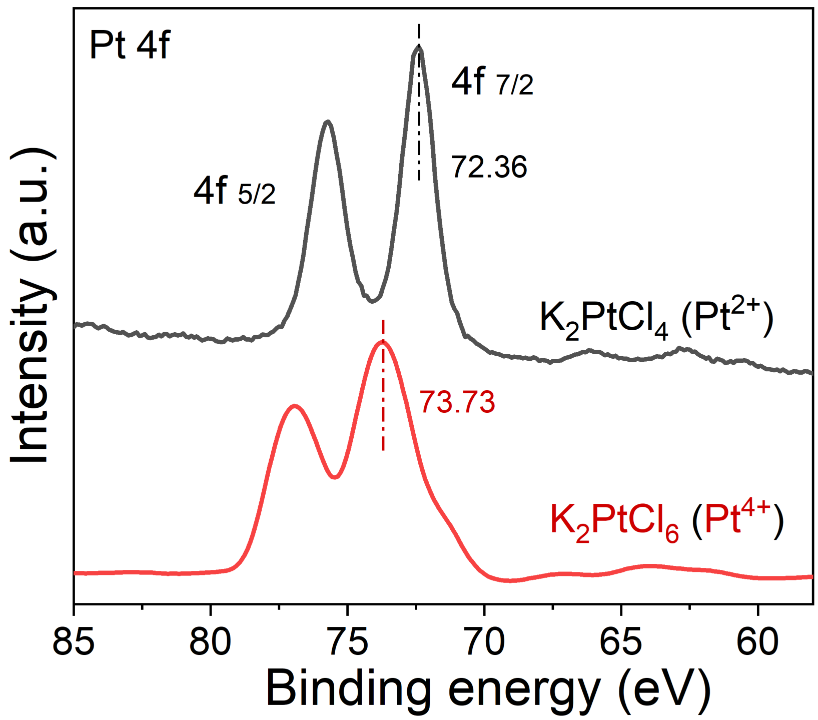


**Supplementary Figure 10.** Pt 4*f* XPS spectra of the $K_2PtCl_4$ ($Pt^{2+}$) and $K_2PtCl_6$ ($Pt^{4+}$) samples.

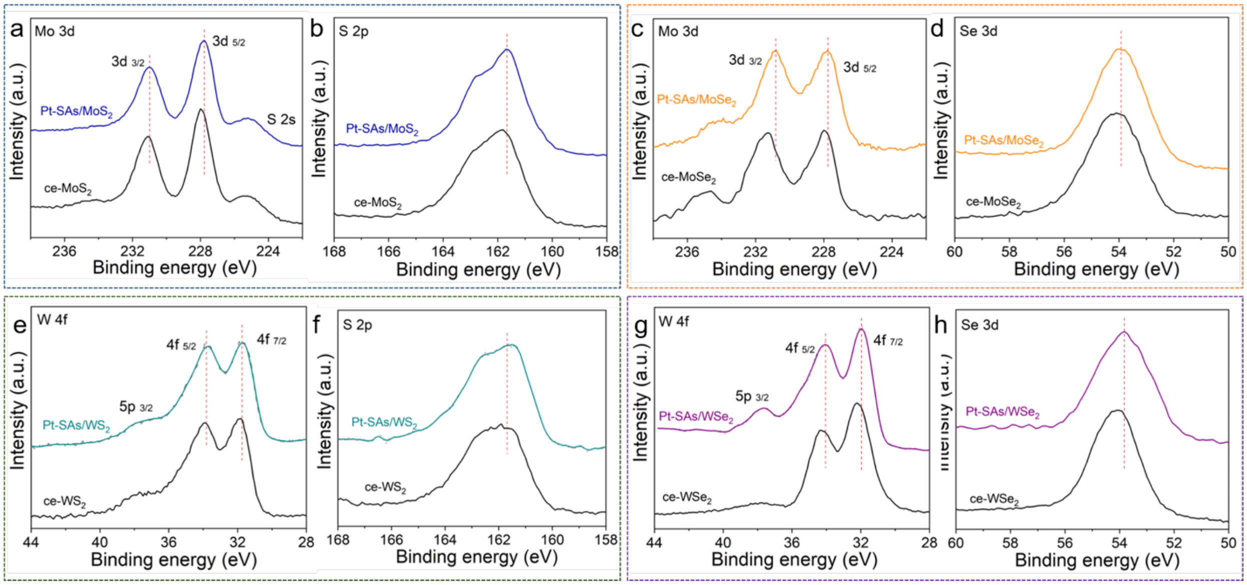


**Supplementary Figure 11.** Mo 3*d* (a) and S 2*p* (b) XPS spectra of Pt-SAs/$MoS_2$ and ce-$MoS_2$. Mo 3*d* (c) and Se 3*d* (d) XPS spectra of Pt-SAs/$MoSe_2$ and ce-$MoSe_2$. W 4*f* (e) and S 2*p* (f) XPS spectra of Pt-SAs/$WS_2$ and ce-$WS_2$. W 4*f* (g) and Se 3*d* (h) XPS spectra of Pt-SAs/$WSe_2$ and ce-$WSe_2$.

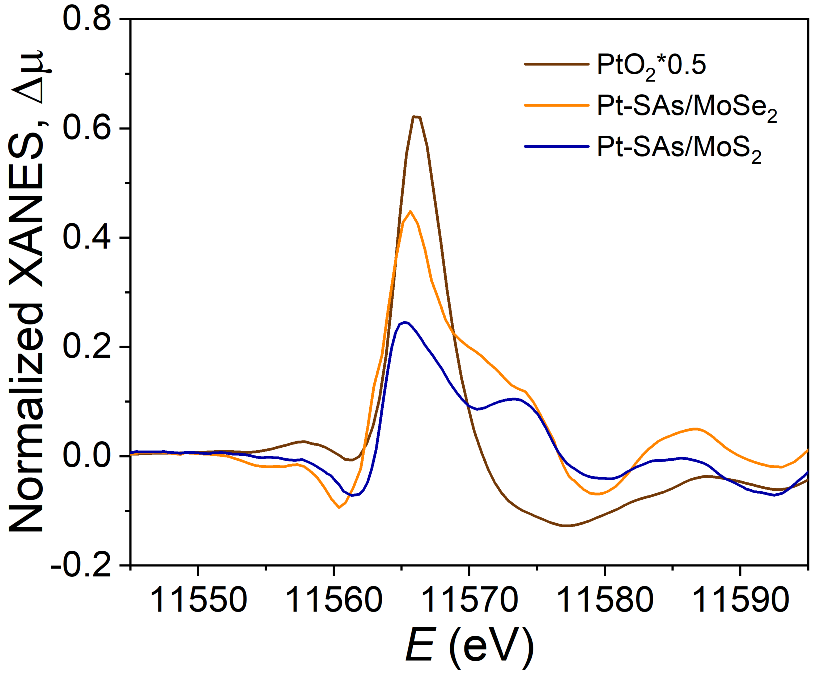


**Supplementary Figure 12.** Normalized ΔXANES spectra for Pt $L_3$-edge using Pt foil as the reference. The oxidation states and the *d*-band hole counts were fitted by integrating the white-line area from 11560 to 11580 eV.

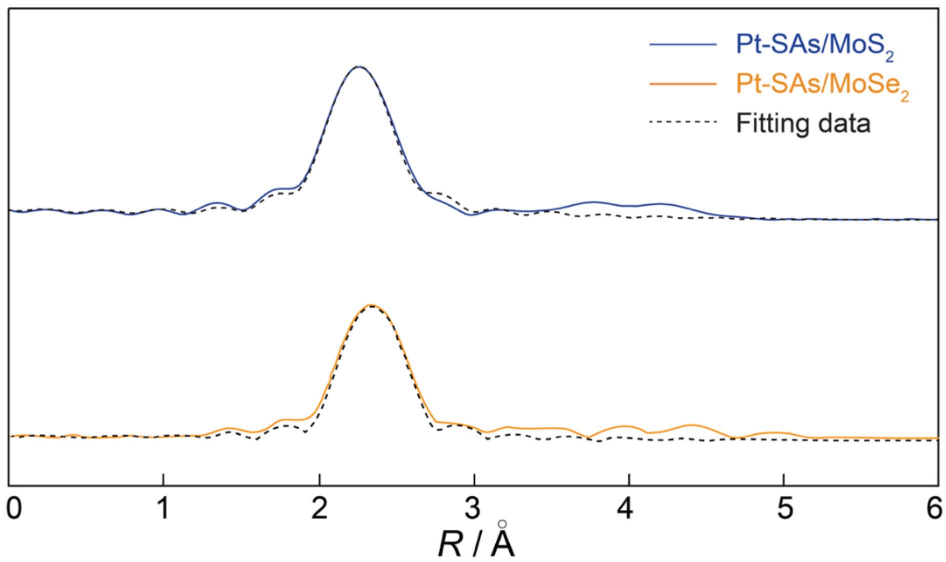


**Supplementary Figure 13.** First-shell fitting for EXAFS profiles of the Fourier transform at Pt $L_3$-edge (fitting parameters shown in Supplementary Tab. 2).

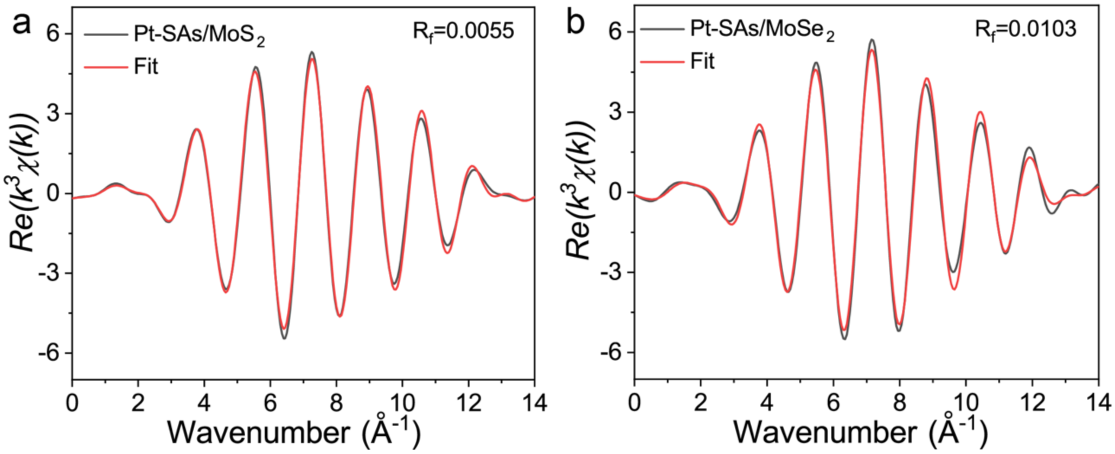


**Supplementary Figure 14.** The fitting curve of $k^3\chi(k)$ oscillations of Pt-SAs/$MoS_2$ (a) and Pt-SAs/$MoSe_2$ (b) using the ARTEMIS module of IFEFFIT.

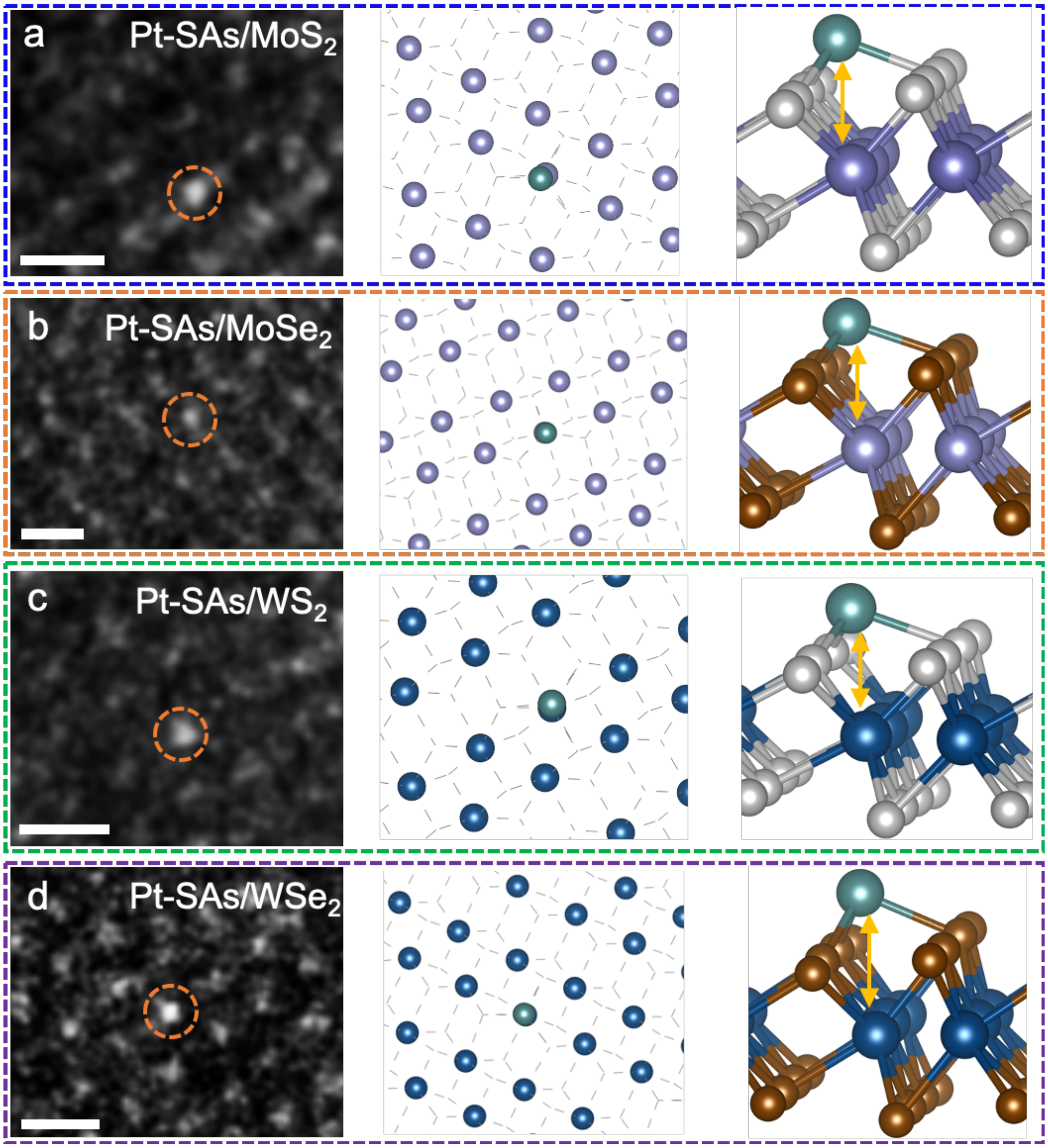


**Supplementary Figure 15.** Magnified HAADF-STEM images of the Pt-SAs/$MoS_2$ (a), Pt-SAs/$MoSe_2$ (b), Pt-SAs/$WS_2$ (c), and Pt-SAs/$WSe_2$ (d) samples (scale bars: 2 nm), and their corresponding top and front views of DFT-optimized structural models (purple: Mo; gray: S; brown: Se; green: Pt; blue: W).

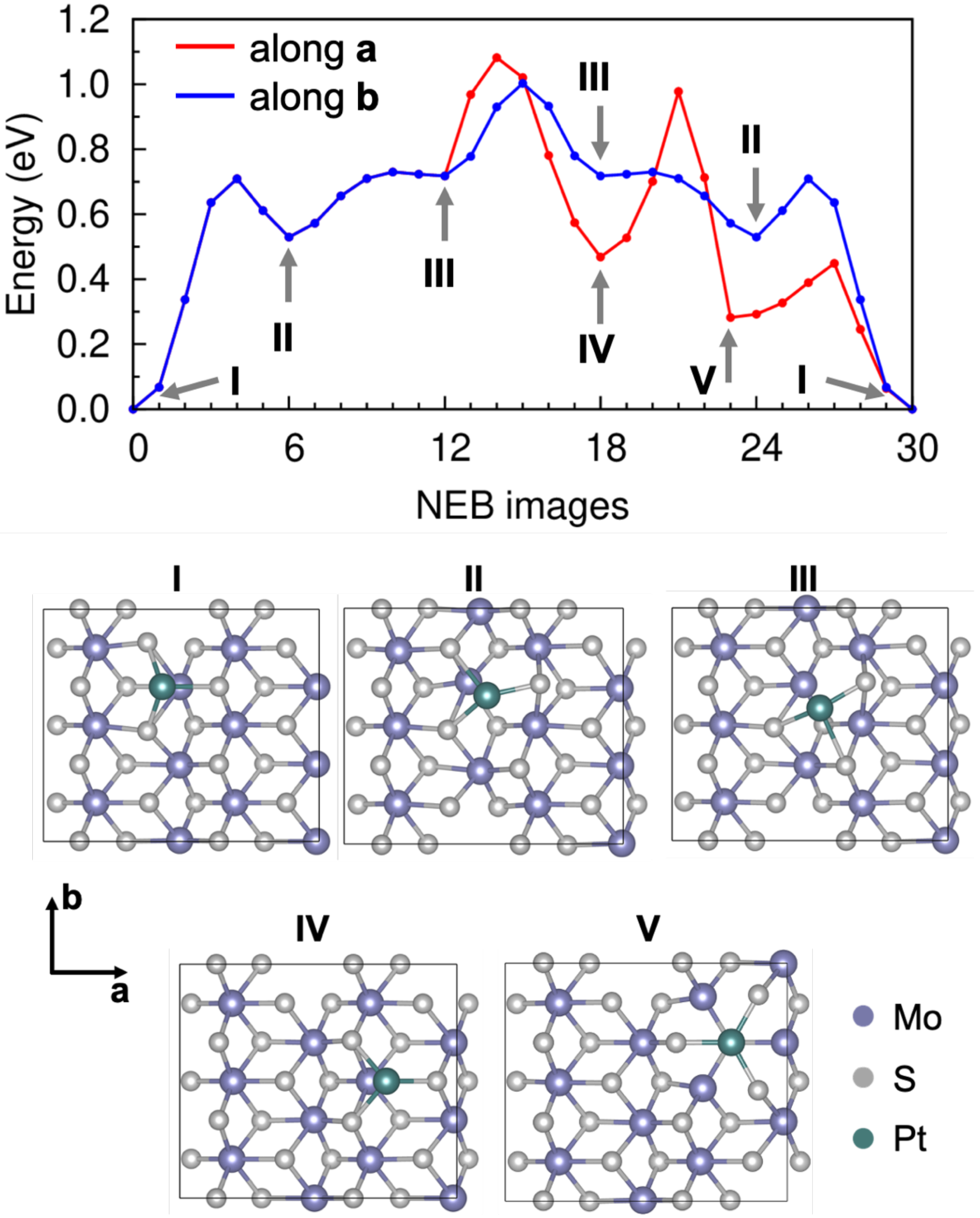


**Supplementary Figure 16.** Energy as a function of reaction coordinate for the diffusion of single-atom Pt between the nearest adsorption sites on $MoS_2$. Model I represents the most stable structure for Pt-SAs/$MoS_2$, while model II~V represent the intermediate metastable states for Pt-SAs/$MoS_2$.

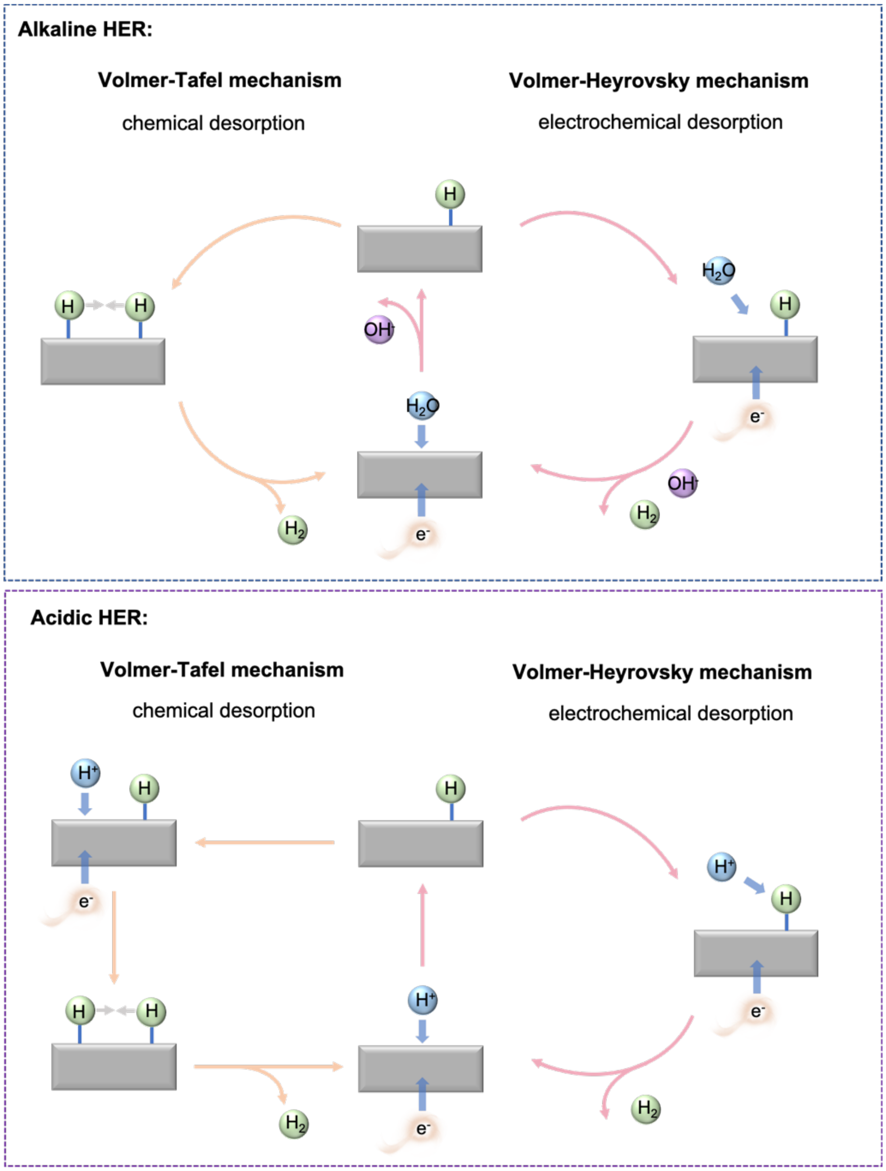


**Supplementary Figure 17.** Schematic representation of the HER mechanism under alkaline and acidic conditions[9]. The alkaline HER proceeds through an initial discharge of water and the formation of adsorbed hydrogen intermediates ($H_{ads}$) during the Volmer step ($H_2O + e^- \rightarrow H_{ads} + OH^-$); followed immediately by either the electrochemical desorption ($H_{ads} + H_2O + e^- \rightarrow H_2 + OH^-$, Heyrovsky step) or the chemical desorption ($H_{ads} + H_{ads} \rightarrow H_2$, Tafel step). The acidic HER pathway is similar, except that $H_{ads}$ is formed by discharge of the hydronium ion ($H_3O^+ + e^- \rightarrow H_{ads} + H_2O$).

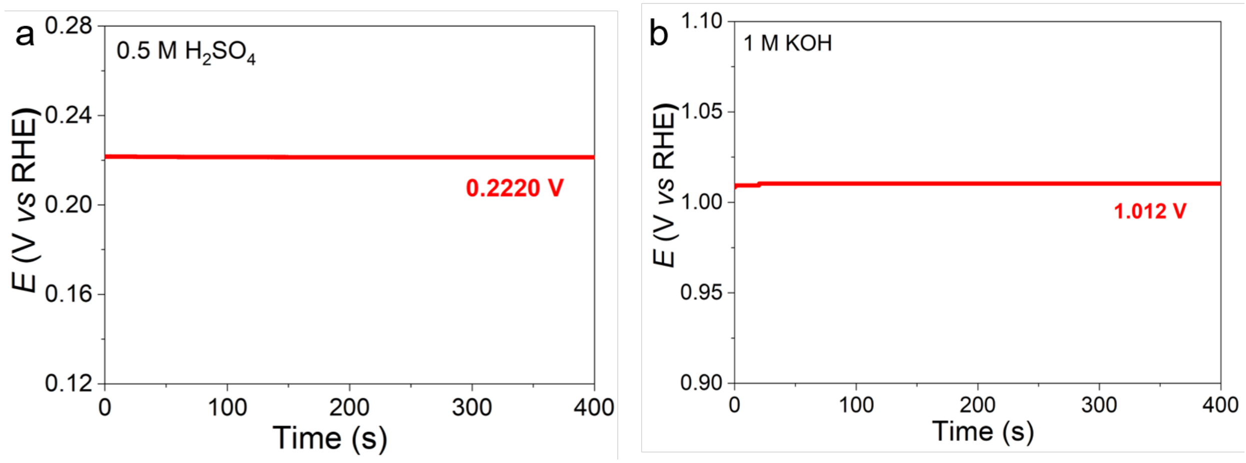


**Supplementary Figure 18.** Calibration of the Ag/AgCl reference electrode in 0.5 M $H_2SO_4$ (a) and 1.0 M KOH (b) solutions, respectively.

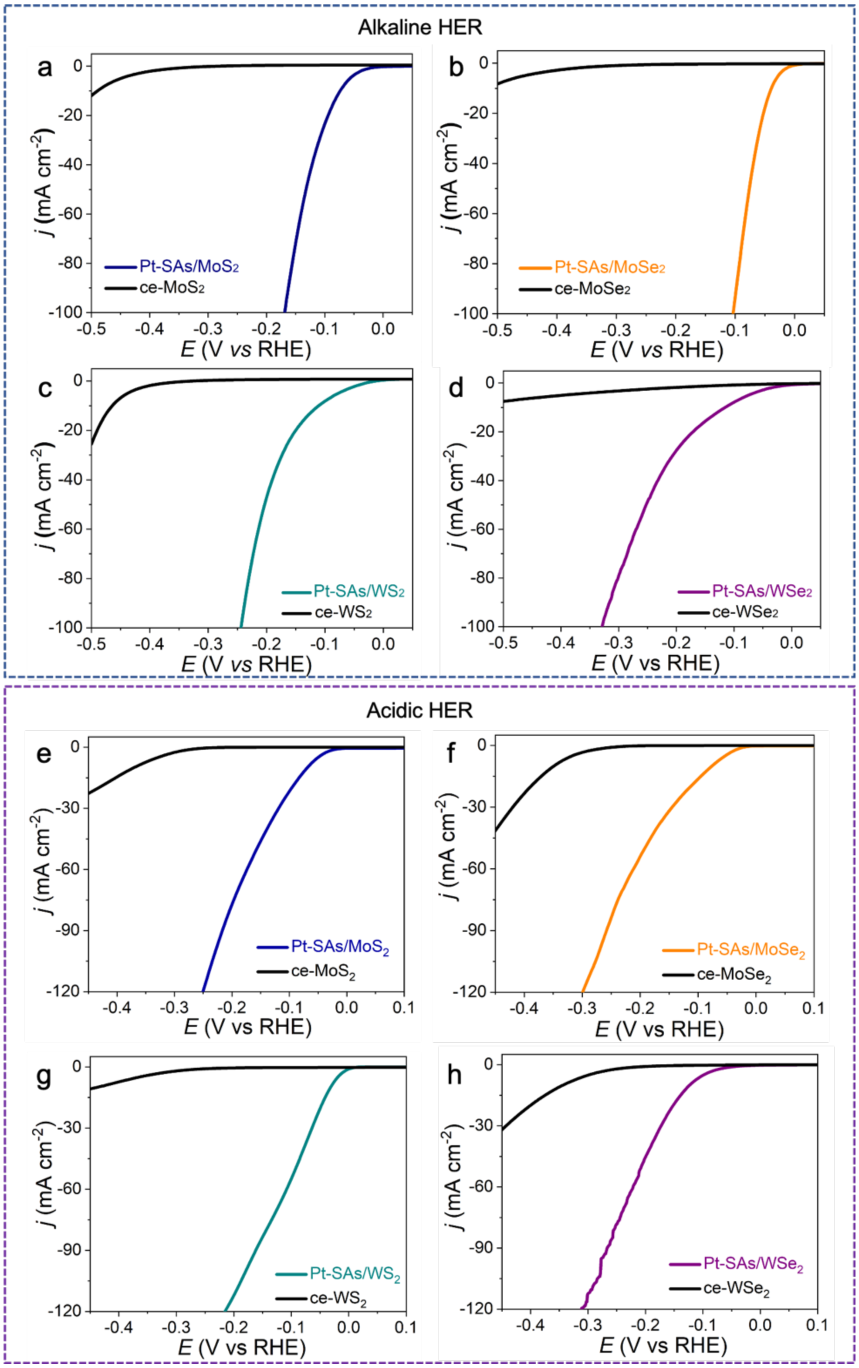


**Supplementary Figure 19.** HER polarization curves for the Pt-SAs/$MoS_2$ (a, e), Pt-SAs/$MoSe_2$ (b, f), Pt-SAs/$WS_2$ (c, g), and Pt-SAs/$WSe_2$ (d, h) samples (colored lines), and their corresponding pure ce-TMDs supports (black lines) in an Ar-saturated 1.0 M KOH (a−d) and 0.5 M $H_2SO_4$ (e−h) at a scan rate of 20 mV $s^{-1}$.

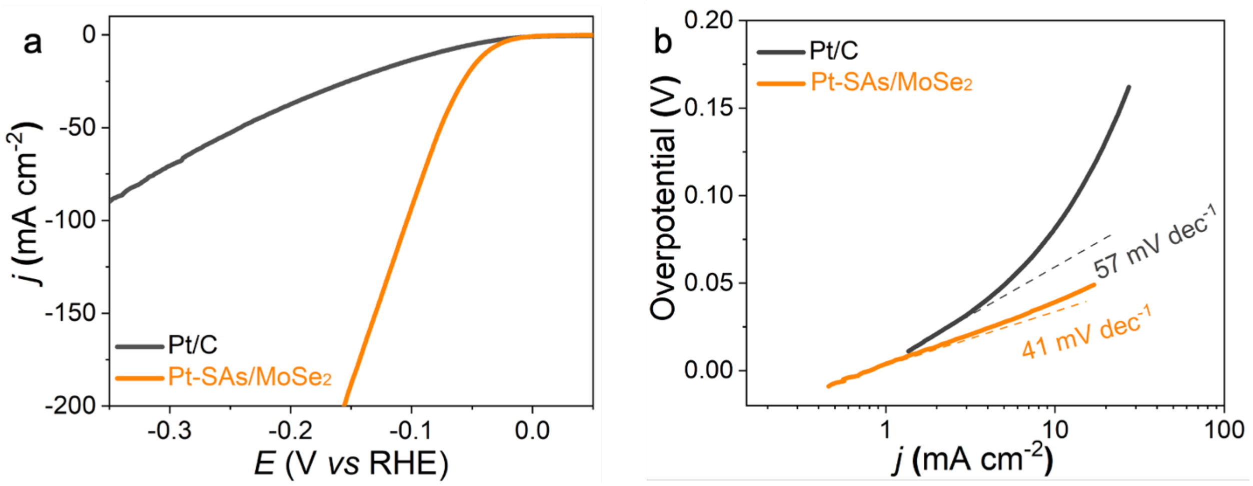


**Supplementary Figure 20.** (a, b) HER polarization curves in 1.0 M KOH for Pt-SAs/$MoSe_2$ and commercial Pt/C at a scan rate of 20 mV $s^{-1}$, and their corresponding Tafel plots derived from the early stage of HER LSV curves.

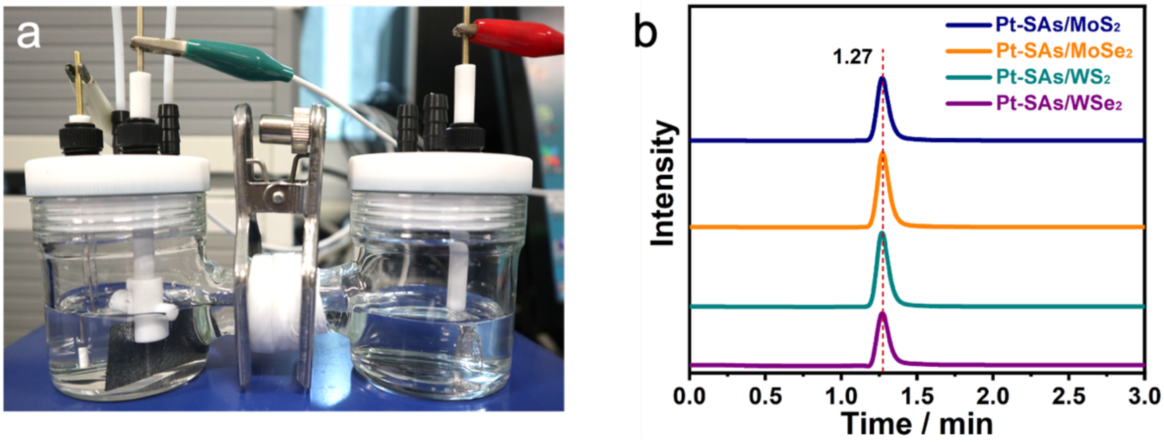


**Supplementary Figure 21.** (a) Electrochemical apparatus used in the gas chromatography (GC) measurements. (b) GC of products catalyzed by Pt-SAs/TMDs in 1.0 M KOH.

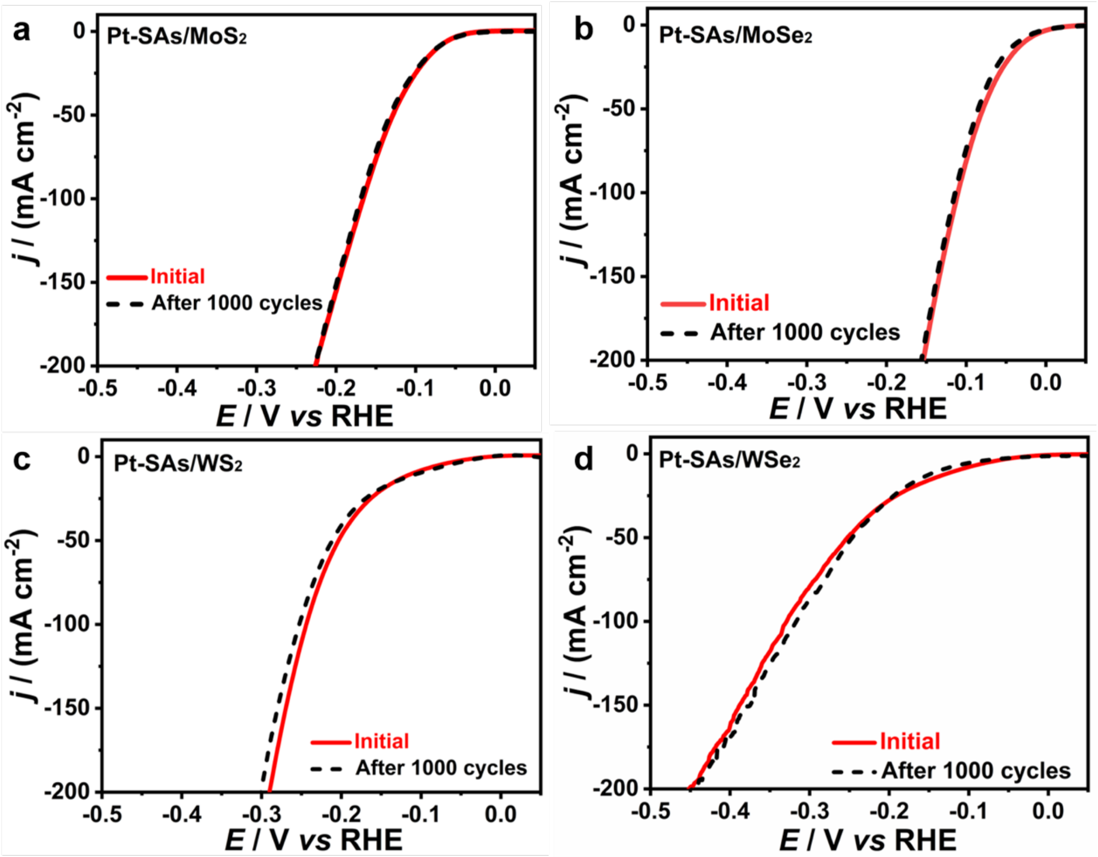


**Supplementary Figure 22.** Stability measurement of Pt-SAs/$MoS_2$ (a), Pt-SAs/$MoSe_2$ (b), Pt-SAs/$WS_2$ (c), and Pt-SAs/$WSe_2$ (d) by potential cycling before (red solid line) and after (black dashed line) 1000 cycles in 1.0 M KOH solution at a scan rate of 20 mV $s^{-1}$.

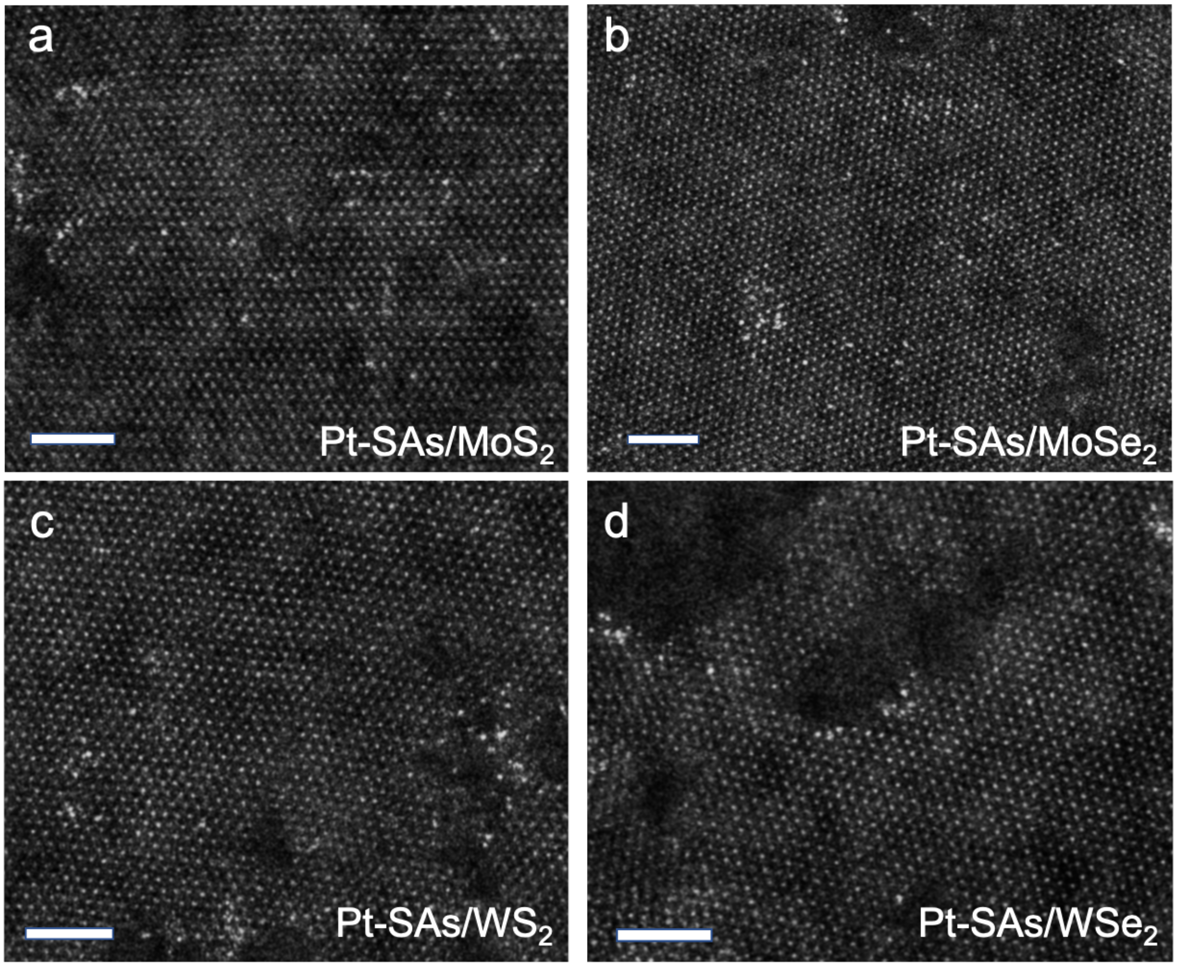


**Supplementary Figure 23.** Atomic-resolution HAADF-STEM images for the Pt-SAs/$MoS_2$ (a), Pt-SAs/$MoSe_2$ (b), Pt-SAs/$WS_2$ (c), and Pt-SAs/$WSe_2$ (d) samples after HER measurements (scale bars: 2 nm).

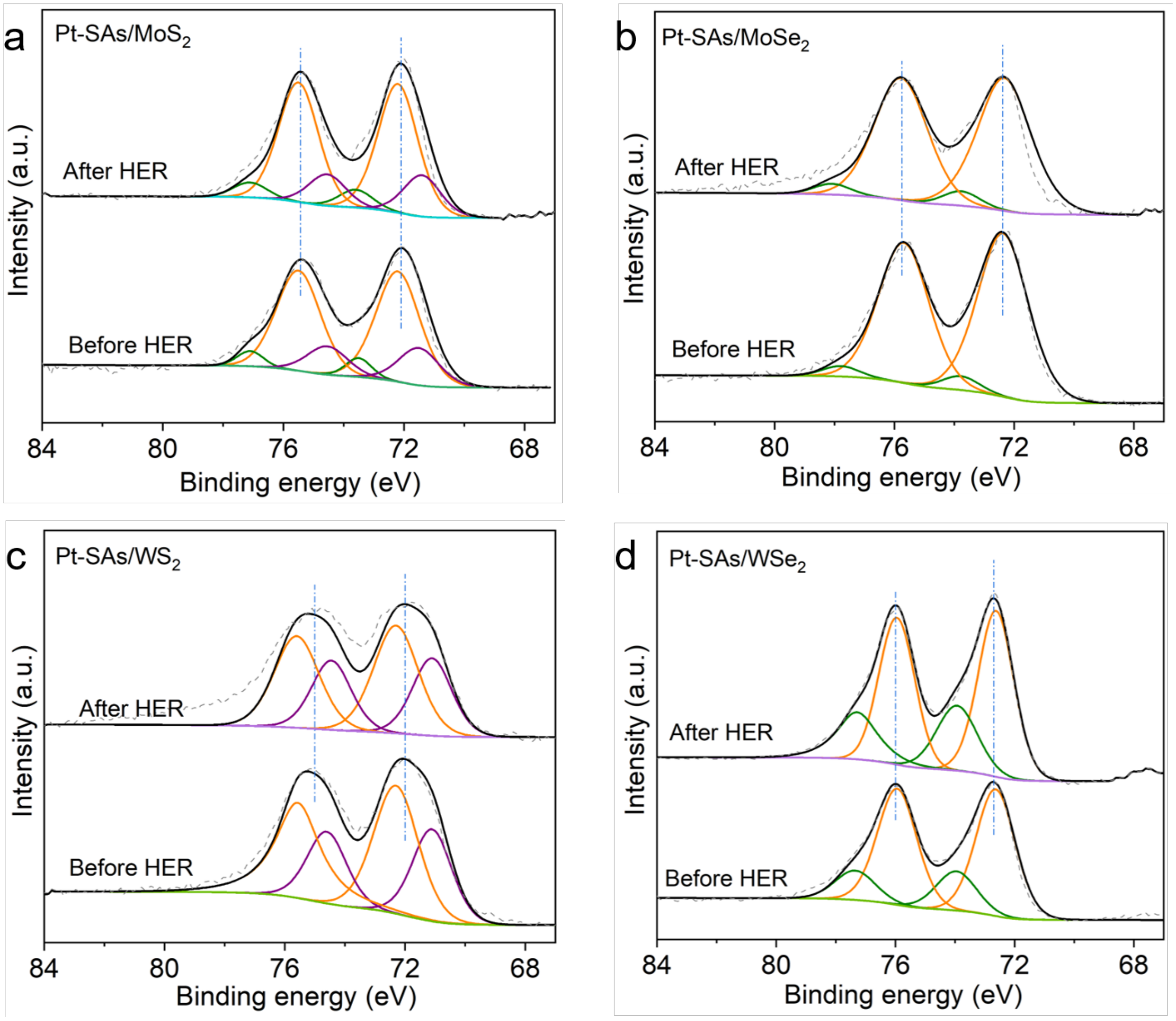


**Supplementary Figure 24.** Pt 4*f* XPS spectra of the Pt-SAs/$MoS_2$ (a), Pt-SAs/$MoSe_2$ (b), Pt-SAs/$WS_2$ (c), and Pt-SAs/$WSe_2$ (d) samples before and after the HER measurements. Quantitative peak deconvolution and integration of XPS analysis showed that after the HER measurements, the average oxidation states of Pt in Pt-SAs/$WS_2$, Pt-SAs/$MoS_2$, Pt-SAs/$MoSe_2$, and Pt-SAs/$WSe_2$ were 1.23, 1.74, 2.13, and 2.60, respectively (Supplementary Tab. 5).

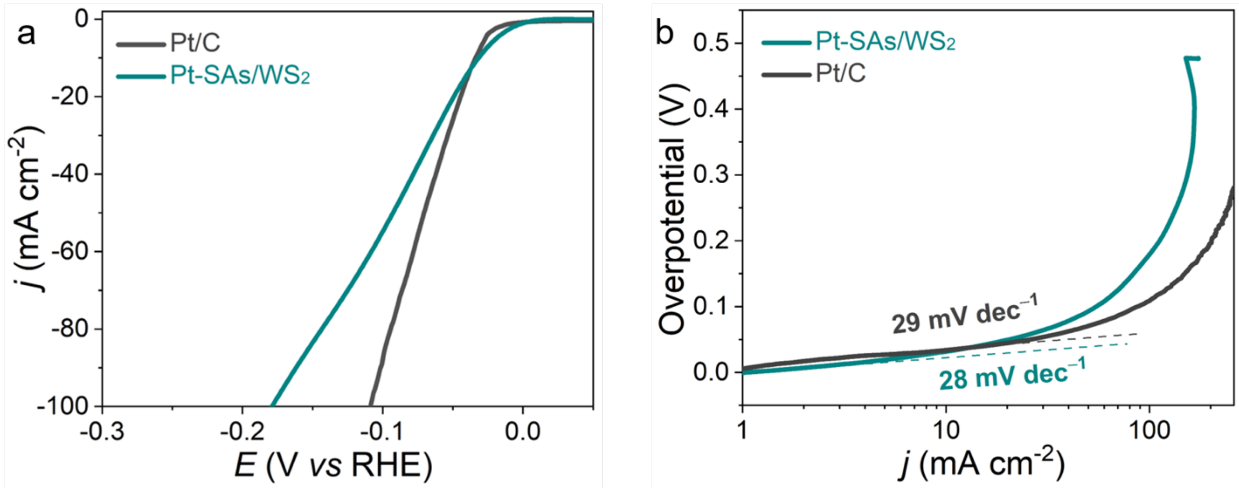


**Supplementary Figure 25.** (a) HER polarization curves in 0.5 M $H_2SO_4$ for Pt-SAs/$WS_2$ and commercial Pt/C at a scan rate of 20 mV $s^{-1}$, and (b) their corresponding Tafel plots derived from the early stage of HER LSV curves.

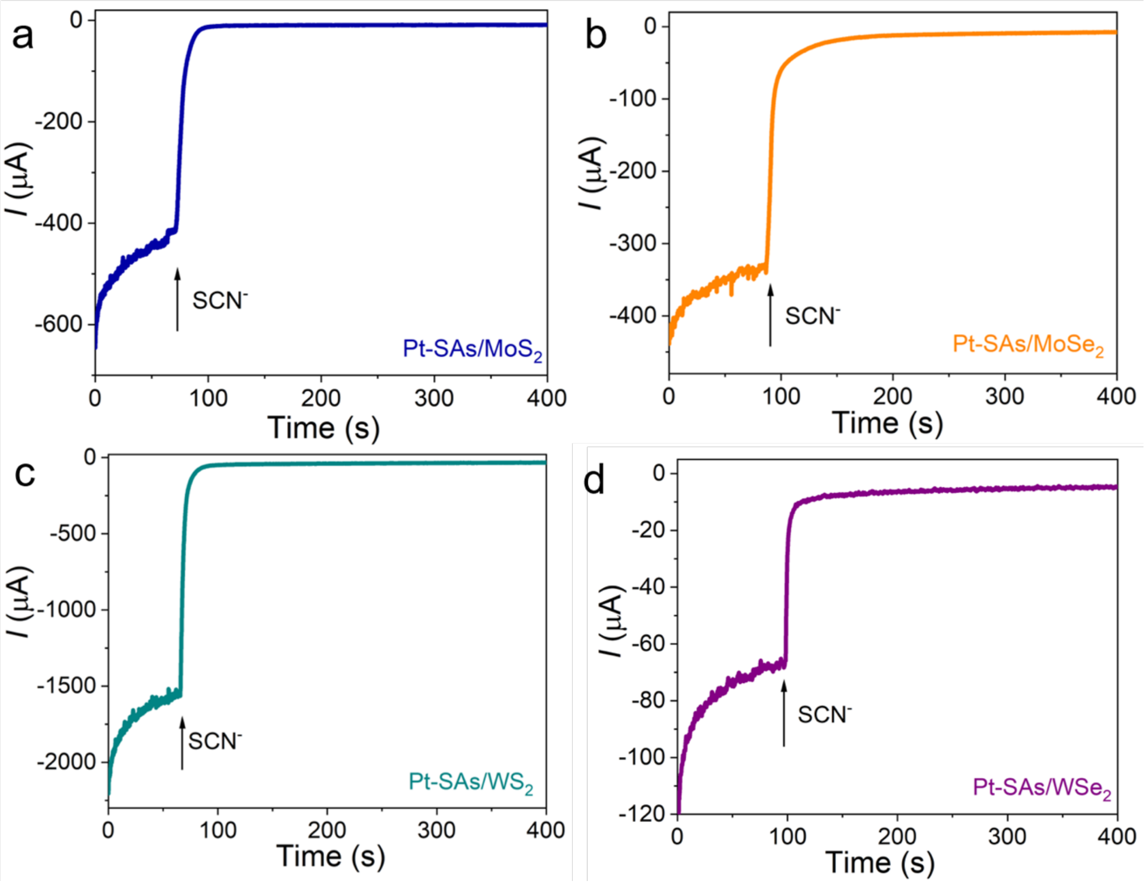


**Supplementary Figure 26.** Current–time curves of the Pt-SAs/$MoS_2$ (a), Pt-SAs/$MoSe_2$ (b), Pt-SAs/$WS_2$ (c), and Pt-SAs/$WSe_2$ (d) samples before and after the addition of 10 mM thiocyanate ions ($SCN^-$) at -0.28 V (vs Ag/AgCl) in 0.5 M $H_2SO_4$.

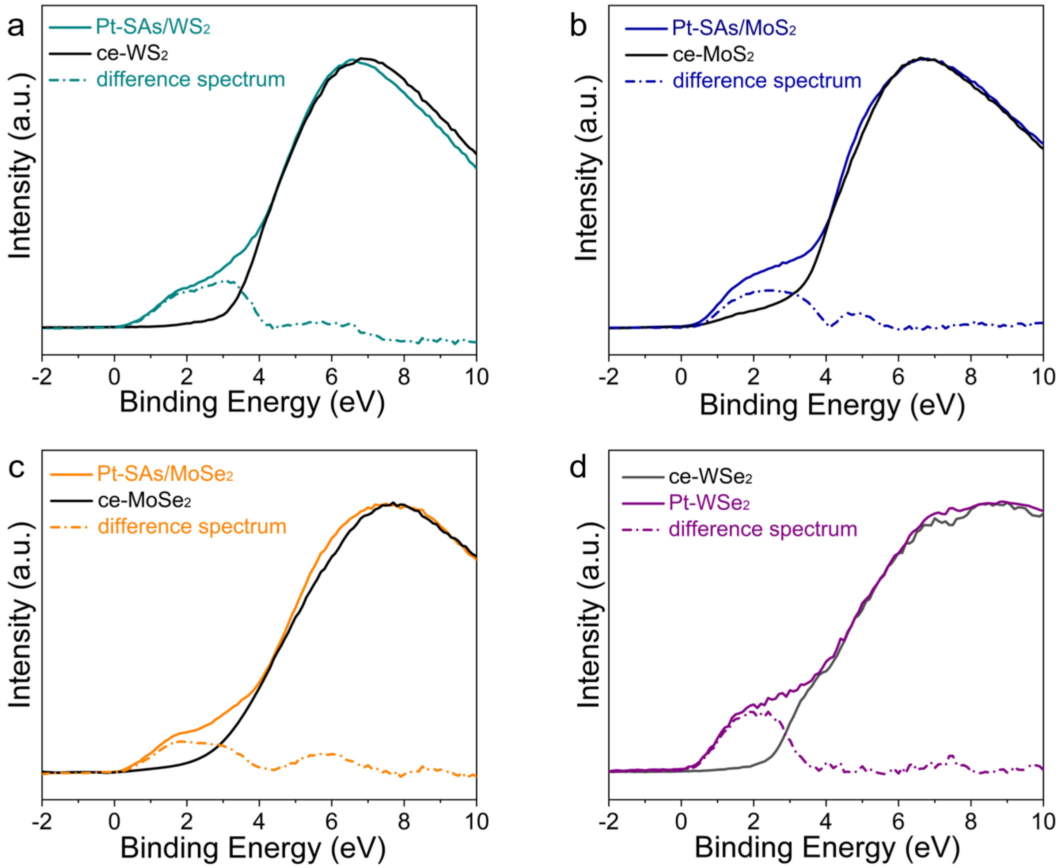


**Supplementary Figure 27.** UPS valence-band spectra of single-atom Pt (colored dash-dotted lines) were obtained by subtraction of the normalized ce-TMDs spectra (black solid lines in (a−d)) from the Pt-SAs/TMDs spectra (colored solid lines in (a−d)).

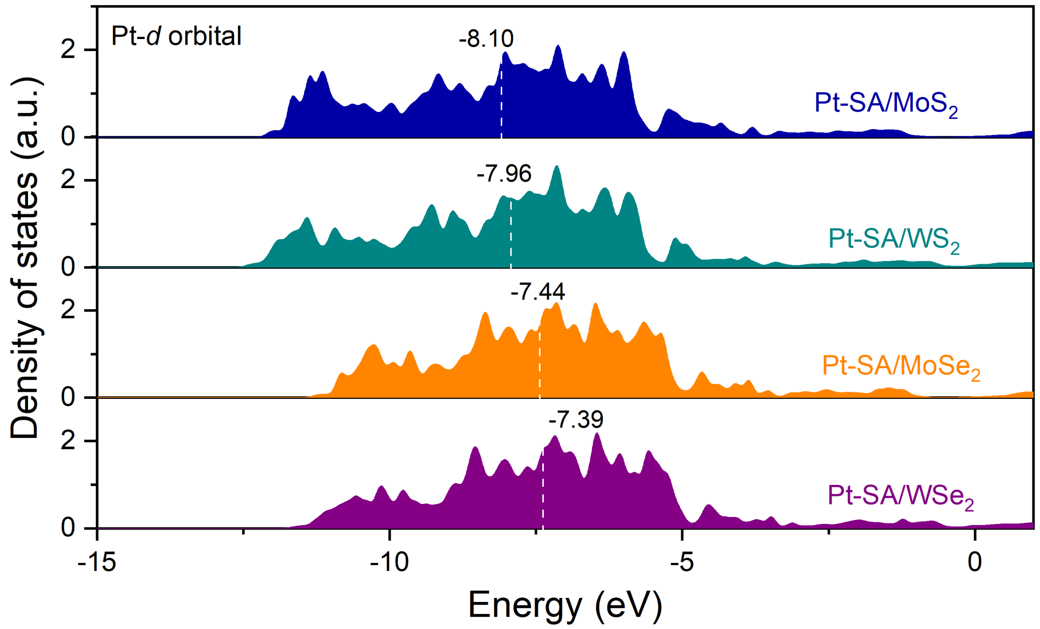


**Supplementary Figure 28.** Density of states projected on Pt-*d* orbitals of Pt-SAs/TMDs. The white dashed lines represent the *d*-band center calculated by DFT. The DFT-calculated positions of *d*-band center follows the order: Pt-SAs/$MoS_2$ (-8.10) < Pt-SAs/$WS_2$ (-7.96) < Pt-SAs/$MoSe_2$ (-7.44) < Pt-SAs/$WSe_2$ (-7.39). Note that the experimental results show the positions of *d*-band center with the order of Pt-SAs/$WS_2$ < Pt-SAs/$MoS_2$ < Pt-SAs/$MoSe_2$ < Pt-SAs/$WSe_2$, whose overall trend is consistent with the theoretical results. The only discrepancy lies in the higher position of Pt-SAs/$MoS_2$. We considered that the discrepancy is mainly caused by the approximations adopted in our calculations for achieving the compromise between accuracy and affordable computational cost. First, the TMD systems used in calculation are simplified to the perfect surface and different from the real situation in the experimental study. Second, the dipole of the whole system is not considered owing to that only one Pt atom was involved in the calculation. Nevertheless, the DFT-calculated results still rationalize the experimental trends (Pt-SAs/$WS_2$ or Pt-SAs/$MoS_2$ < Pt-SAs/$MoSe_2$ < Pt-SAs/$WSe_2$), which is of great reference value to our work.

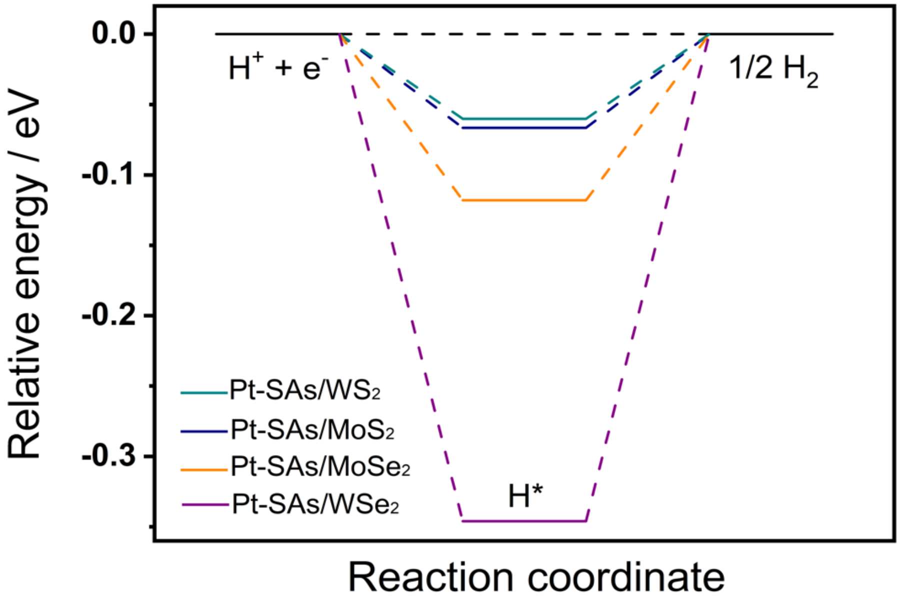


**Supplementary Figure 29.** Calculated free energy ($\Delta G$) barrier diagram of HER at the equilibrium potential at pH 0 for various catalysts. The free energies of H adsorption on Pt-SAs/$WS_2$, Pt-SAs/$MoS_2$ Pt-SAs/$MoSe_2$, and Pt-SAs/$WSe_2$ are -0.060, -0.067, -0.118, and -0.343 eV, respectively, which agree well with the H adsorption ability predicted by the *d*-band theory.

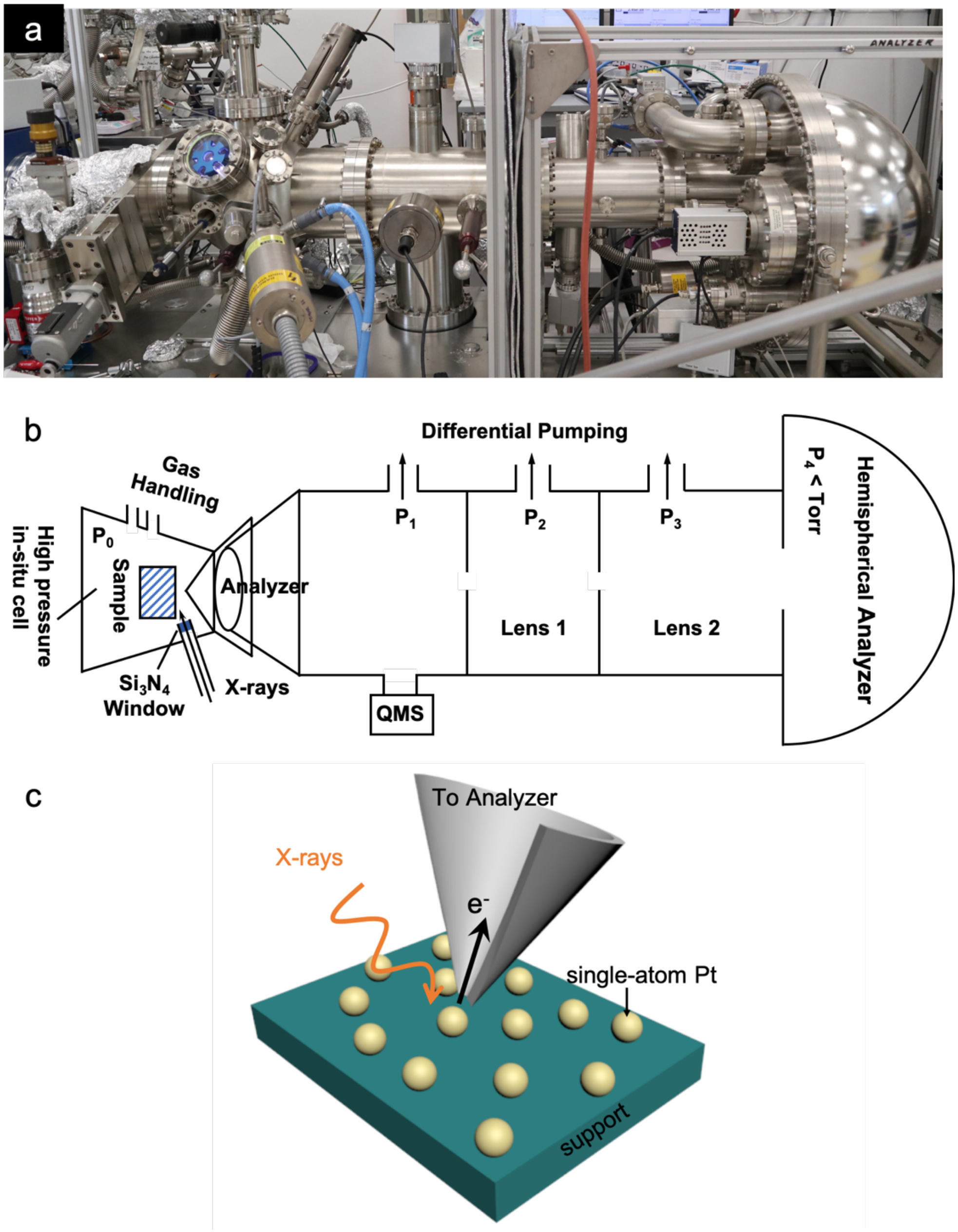


**Supplementary Figure 30.** Photograph (a) and schematic diagram (b) of the NAP-XPS system. (c) Schematic illustration of the combined NAP-XPS with hydrogen absorption measurements for Pt-SAs/TMDs samples.

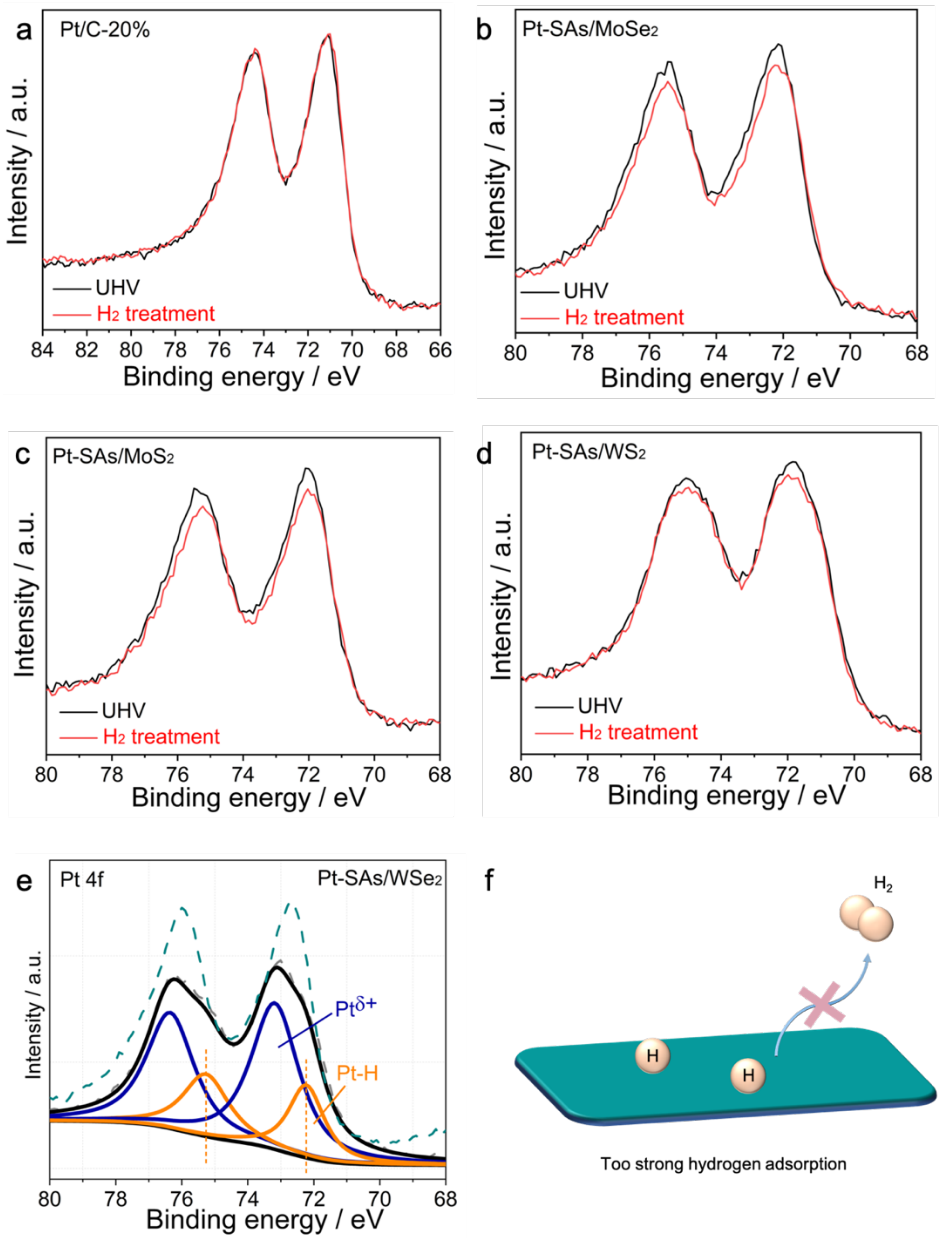


**Supplementary Figure 31.** (a~e) NAP-XPS spectra of Pt 4*f* in different Pt-based samples carried out at room temperature under UHV and 0.5 mbar of $H_2$ atmosphere. New obvious peak at *ca.* 72.2 eV emerged in the Pt 4*f* peak of Pt-SAs/$WSe_2$ (e), which could be ascribed to the formation of strong Pt−H bond, whereas no obvious changes were observed in commercial Pt/C (a) and three other Pt-SAs samples (b~d). (f) Schematic illustrating that too strong hydrogen adsorption results in the slow release of active sites, and hence the sluggish overall HER rate.

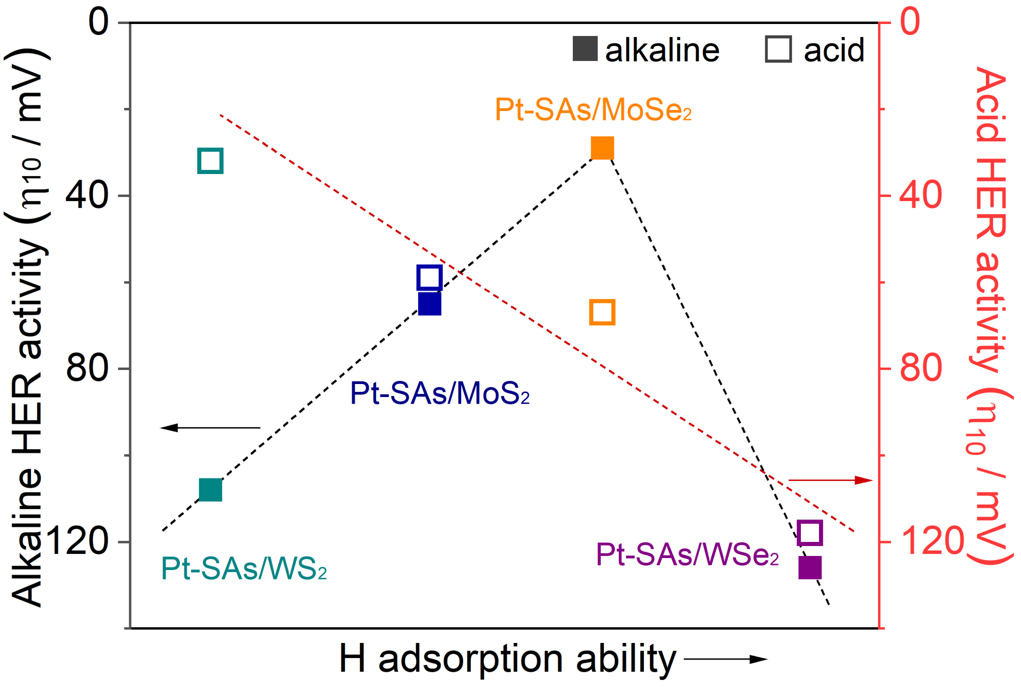


**Supplementary Figure 32.** Relationship between H adsorption ability (represented according to the position of the *d*-band center) of Pt-SAs/TMDs samples and their corresponding acid/alkaline HER activity (represented by the overpotential at a current density of 10 mA $cm^{-2}$).

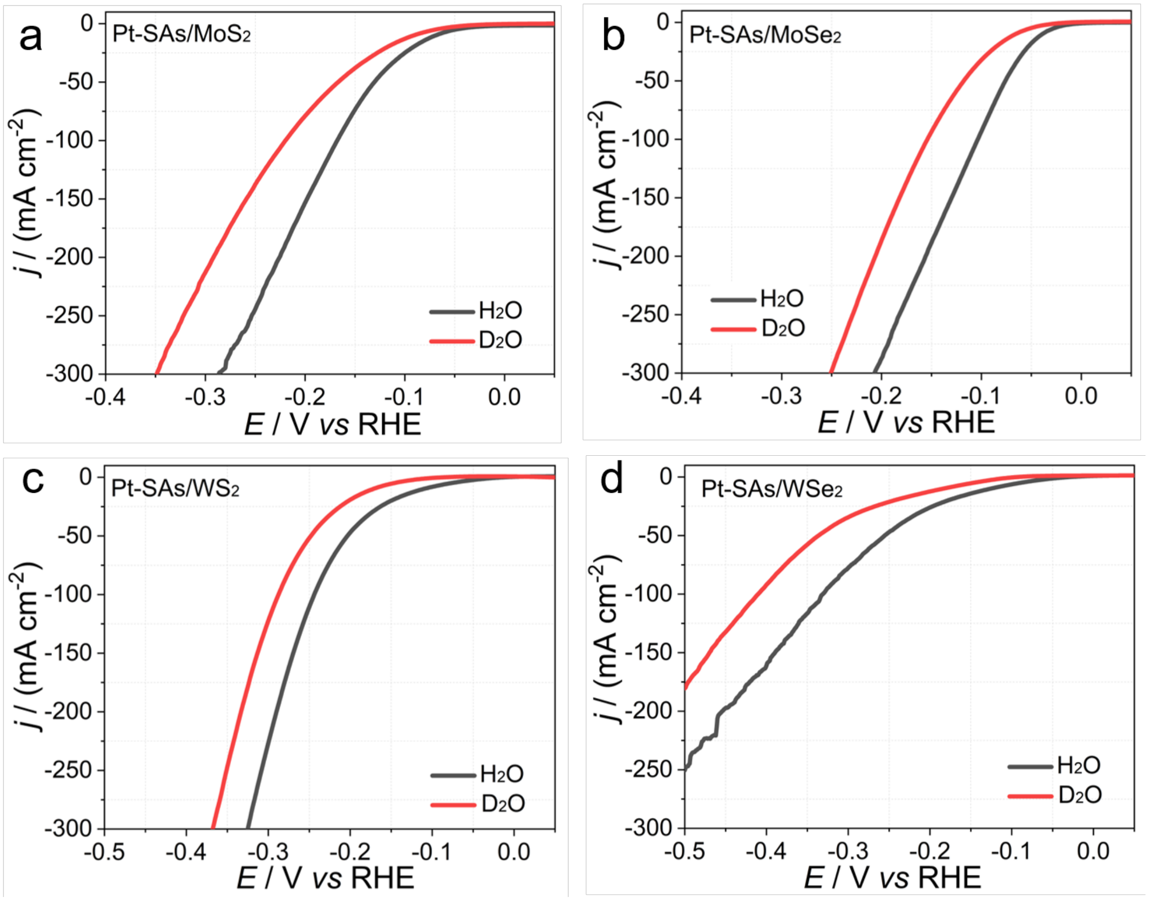


**Supplementary Figure 33.** HER polarization curves obtained on the Pt-SAs/$MoS_2$ (a), Pt-SAs/$MoSe_2$ (b), Pt-SAs/$WS_2$ (c), and Pt-SAs/$WSe_2$ (d) samples in 1.0 M $D_2O$/KOD (red lines) or $H_2O$/KOH (black lines) solution at a scan rate of 20 mV $s^{-1}$.

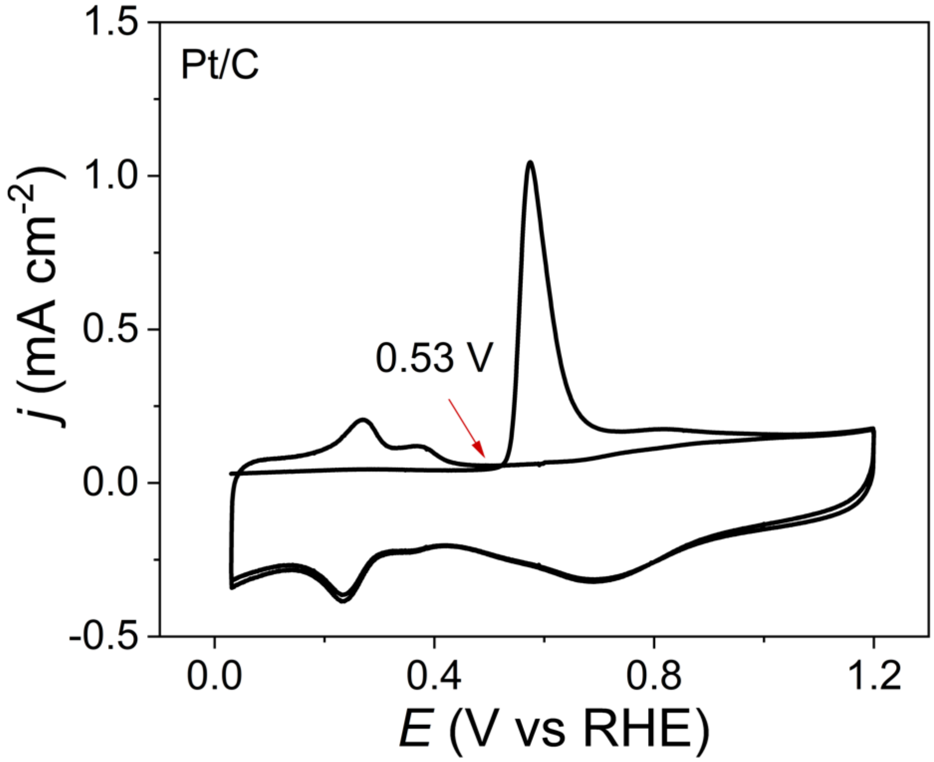


**Supplementary Figure 34.** CO stripping voltammetry of commercial Pt/C at a scan rate of 50 mV $s^{-1}$. The red arrow represents the CO oxidation potentials.

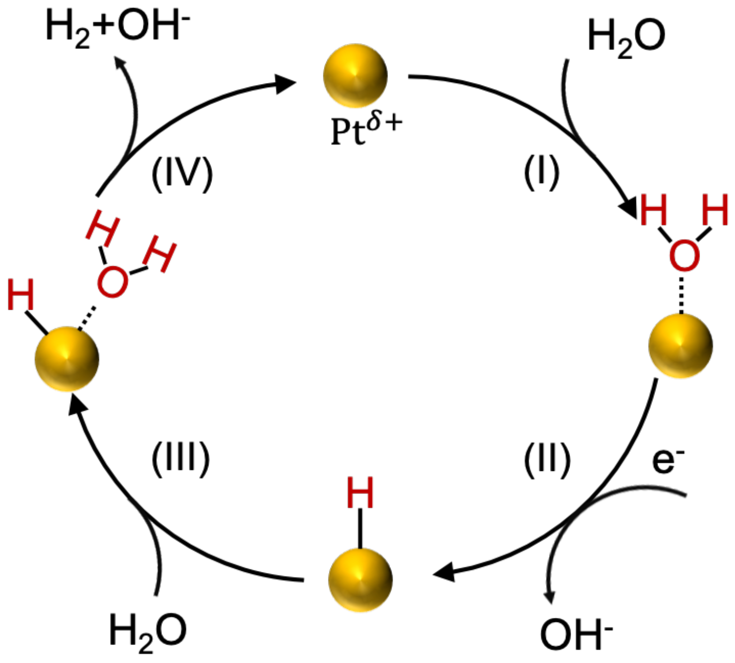


**Supplementary Figure 35.** Schematic illustration of water dissociation and hydrogen recombination/desorption on Pt-SAs/TMDs in alkaline HER. First, $H_2O$ molecule is absorbed onto Pt atom (step I), and then dissociates into adsorbed H (H*) on Pt and $OH^-$ (step II). Subsequently, another adjacent proton derived from a $H_2O$ molecule on the same Pt atom reacts with the first H* to release $H_2$ molecule (steps III and IV).

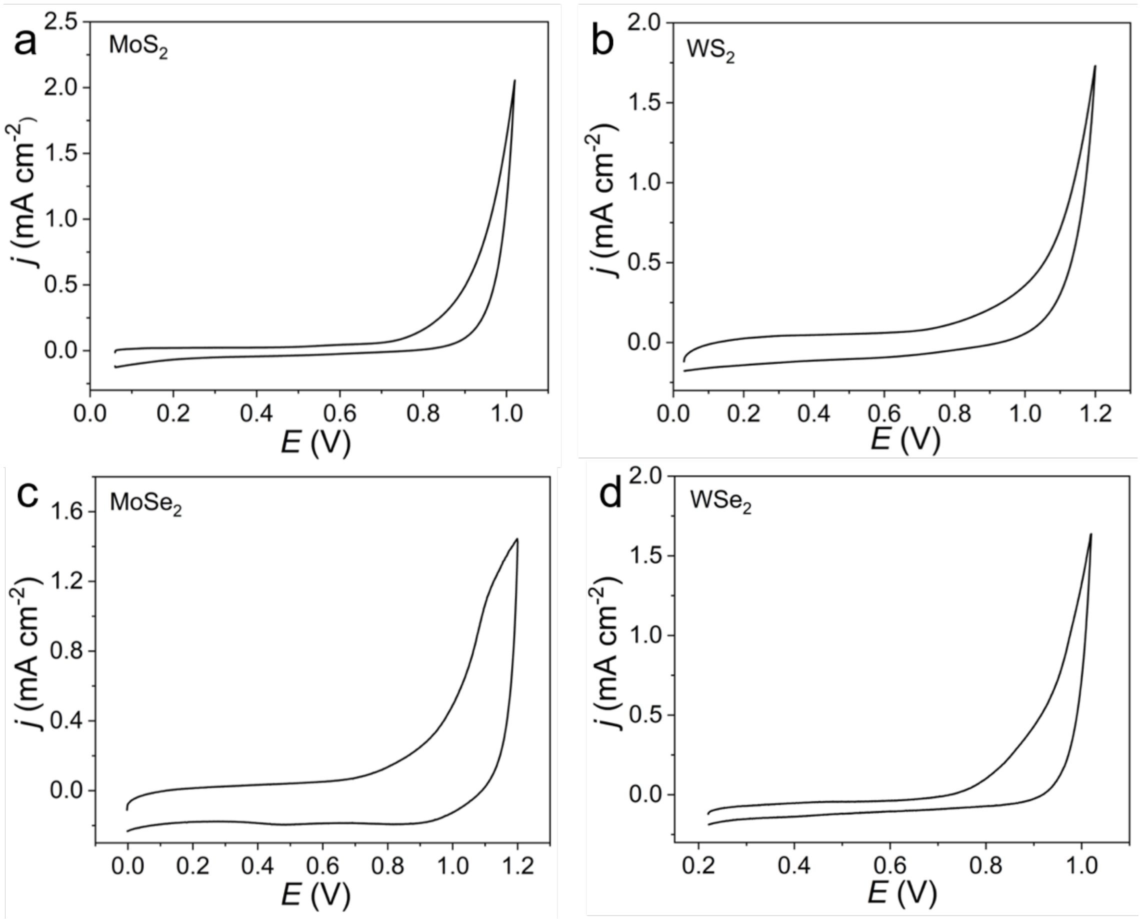


**Supplementary Figure 36.** CVs of ce-$MoS_2$ (a), ce-$WS_2$ (b), ce-$MoSe_2$ (c), and ce-$WSe_2$ (d) in 1.0 M KOH solution at a scan rate of 50 mV $s^{-1}$. Oxidation currents starting from ~0.7 V (vs RHE) were attributable to the transitions from TMDs→$MoO_x$ or $WO_x$.

## Supplementary Tables

**Supplementary Table 1. Average oxidation state of Pt calculated from the XPS spectra of the Pt-SAs/TMDs catalysts.**

| Sample | Binding energy of Pt $4f_{7/2}$ (eV) | Content (%) | Average oxidation state of Pt from XPS results[a] |
|---|---|---|---|
| Pt-SAs/$WS_2$ | 72.30 ($Pt^{II}$) | 62.2 | 1.24 |
| | 71.14 ($Pt^0$) | 37.8 | |
| Pt-SAs/$MoS_2$ | 73.60 ($Pt^{IV}$) | 7.1 | 1.71 |
| | 72.20 ($Pt^{II}$) | 70.9 | |
| | 71.35 ($Pt^0$) | 22.0 | |
| Pt-SAs/$MoSe_2$ | 73.78 ($Pt^{IV}$) | 5.7 | 2.11 |
| | 72.37 ($Pt^{II}$) | 94.3 | |
| Pt-SAs/$WSe_2$ | 73.95 ($Pt^{IV}$) | 30.6 | 2.61 |
| | 72.62 ($Pt^{II}$) | 69.4 | |

[a] For XPS analysis, the average oxidation state of Pt is calculated according to the below equation (S-2)[4]:

$$Pt_{Average\ oxidation\ state} = Content\%_{(Pt(IV))} \times 4 + Content\%_{(Pt(II))} \times 2 + Content\%_{(Pt(0))} \times 0 \quad \text{(S-2)}$$

**Supplementary Table 2. Fitting parameters of the Fourier transform of the first shell of the EXAFS spectra.**

| Sample | Scatter | CN | $R$ / Å | $\sigma^2$ / Å$^2$ | $\Delta E_0$ / eV | r factor |
|---|---|---|---|---|---|---|
| Pt-SAs/$MoS_2$ | Pt–S | 3.2 | 2.27 | 0.0050 | 2.08 | 0.0055 |
| Pt-SAs/$MoSe_2$ | Pt–Se | 3.5 | 2.35 | 0.0054 | 3.37 | 0.0103 |

CN, coordination number; $R$, average bond distance; $\sigma^2$, Debye–Waller factor; $\Delta E_0$, inner potential correction; r factor reflects the goodness of the fit (<2%).

**Supplementary Table 3. Comparison of HER activity for Pt-SAs/TMDs in alkaline solution with the state-of-the-art single-atom catalysts reported previously.**

| Catalyst | loading | Electrolyte | $\eta$ (mV) | Tafel slope (mV $dec^{-1}$) | $\eta_{10}$ (mV) | TOF ($s^{-1}$) | Reference |
|---|---|---|---|---|---|---|---|
| Pt-SAs/$MoSe_2$ | 4.7 wt% | 1.0 M KOH | ~0 | 34 | 29 | 6.21@50 mV | This work |
| Pt-SAs/$MoS_2$ | 5.1 wt% | 1.0 M KOH | ~0 | 50 | 65 | 1.02@50 mV | This work |
| Pt-SAs/$WS_2$ | 4.1 wt% | 1.0 M KOH | ~0 | 55 | 108 | 0.56@50 mV | This work |
| Pt-SAs/$WSe_2$ | 4.9 wt% | 1.0 M KOH | ~0 | 59 | 126 | 0.52@50 mV | This work |
| Pt@PCM | 0.53 wt% | 1.0 M KOH | ~50 | 73.6 | 139 | NA | *Sci. Adv.* 2018, 4, 6657 |
| $Pt_1$@Fe-N-C | 2.1 wt% | 1.0 M KOH | ~50 | / | 108 | NA | *Adv. Energy Mater.* 2018, 8, 1701345 |
| Co-NG | 0.57 at% | 1 M NaOH | ~100 | / | 280 | NA | *Nat. Commun.* 2015, 6, 8668 |
| RuAu SAAs | 15.35 at% | 1.0 M KOH | ~0 | 37 | 24 | 2.18@50 mV | *Adv. Energy Mater.* 2019, 1803913 |
| $Ni_{SA}$-$MoS_2$/CC | 1.8 at% | 1.0 M KOH | ~0 | 75 | 95 | NA | *Nano Energy* 2018, 53, 458 |
| W-SAC | 1.21 wt% | 0.1 M KOH | ~0 | 53 | 85 | 6.35@120 mV | *Adv. Mater.* 2018, 30, 1800396 |
| $Mo_1N_1C_2$ | 1.32 wt% | 0.1 M KOH | 13 | 90 | 132 | 1.46@150 mV | *Angew. Chem. Int. Ed.* 2017, 56, 16086 |
| Co-Ru NSs | 6.0 at% | 1.0 M KOH | ~0 | 29 | 13 | 6.93@60 mV | *Nat. Commun.* 2018, 9, 4958 |
| PtSA-NT-NF | 1.76 wt% | 1.0 M KOH | ~0 | NA | 20 | NA | *Angew. Chem. Int. Ed.* 2017, 56, 13694 |
| $Co_1$/PCN | 0.3 wt% | 1.0 M KOH | ~0 | 52 | 89 | 5.98@100 mV | *Nat. Catal.* 2019, 2, 134 |

| | | | | | | | |
|---|---|---|---|---|---|---|---|
| Ru-NC | 4.0 at% | 0.1 M KOH | ~0 | 14 | 47 | NA | *Nat. Commun.* 2019, 10, 631 |

Note that the red rows represent the alkaline HER activity of Pt-SAs/TMDs in this study.

**Supplementary Table 4. Comparison of HER activity for Pt-SAs/$MoSe_2$ in alkaline solution with the state-of-the-art Pt-based catalysts reported previously.**

| Catalyst | Loading ($\mu g_{Pt}\ cm_{disk}^{-2}$) | Electrolyte | Current density (mA $cm^{-2}$) | $\eta_{10}$ (mV) | Tafel slope (mV $dec^{-1}$) | Reference |
|---|---|---|---|---|---|---|
| Pt-SAs/$MoSe_2$ | 2.89 | 1 M KOH | 99.47@100 mV | 29 | 34 | This work |
| BPed-Pt/GR | 14.28 | 1 M KOH | 154.7@100 mV | 21 | 46.9 | Angew. Chem. Int. Ed., 2019, 58, 19060 |
| PtRu NCs/BP | 14.8 | 1 M KOH | 88.5@70 mV | 22 | 19 | ACS Catal. 2019, 9, 10870 |
| $Pt_{6.2}$Ni-S NWs | 15.31 | 1 M KOH | 75.3@70 mV | 24 | NA | Angew. Chem. Int. Ed. 2018, 57, 11678 |
| $Pt_3Ni_3$-NWs | 15 | 1 M KOH | 39.7@70 mV | 40 | NA | Angew. Chem. 2016, 128, 13051 |
| SA In-Pt NWs/C | 5.1 | 1 M KOH | ~30@90 mV | 46 | 32.4 | Adv. Funct. Mater. 2020, 30, 2004310 |
| $Ni(OH)_2$ modified Pt | NA | 0.1 M KOH | ~9.5 | ~75 | 75±5 | Angew. Chem., Int. Ed., 2012, 51, 12495 |
| PtNi-O/C | 5.1 | 1 M KOH | 36.9@70 mV | 40 | 78.8 | J. Am. Chem. Soc. 2018, 140, 9046 |
| Pt-Ni ASs | 17 | 1 M KOH | ~42@70 mV | 27.7 | 27 | Adv. Mater. 2018, 30, 1801741 |
| $Pt_3Ni_2/NiS_x$ NWs | 15.31 | 1 M KOH | 37.2@70 mV | 42 | NA | Nat. Commun. 2017, 8, 14580 |
| Pt–$Co(OH)_2$/CC | 390 | 1 M KOH | ~70@100 mV | 32 | 70 | ACS Catal. 2017, 7, 7131 |
| PtCo–Co/TiM | 43 | 1 M KOH | 46.5@70 mV | 28 | 35 | Nanoscale, 2018, 10, 12302 |
| $Ni_3N$/Pt | ~300 | 1 M KOH | ~35@70 mV | 50 | 36.5 | Adv. Energy Mater. 2016, 1601390 |
| PtNi–Ni NA/CC | 69.3 | 0.1 M KOH | ~20@60 mV | 38 | 42 | Inorg. Chem. Front., 2018, 5, 1365 |

| | | | | | | |
|---|---|---|---|---|---|---|
| *hcp*-Pt-Ni | 8 | 0.1 M KOH | ~24.2@70 mV | ~67 | 74 | Nat. Commun., 2017, 8, 15131 |
| $Ni(OH)_2$/Pt(111) surface | NA | 0.1 M KOH | ~2.2 | ~138 | ~100-130 | Science, 2011, 334, 1256 |
| Pt NWs/SL-$Ni(OH)_2$ | 16 | 1 M KOH | 10.9 | ~70 | NA | Nat. Commun. 2015, 6, 6430 |
| | | 0.1 M KOH | 26.6 | ~48 | NA | |

Note that the red rows represent the alkaline HER activity of Pt-SAs/$MoSe_2$ in this study.

**Supplementary Table 5. Average oxidation state of Pt calculated from the XPS spectra of the Pt-SAs/TMDs catalysts after HER measurements.**

| Sample | Binding energy of Pt $4f_{7/2}$ (eV) | Content (%) | Average oxidation state of Pt after HER measurement |
|---|---|---|---|
| Pt-SAs/$WS_2$ | 72.30 ($Pt^{II}$) | 61.3 | 1.23 |
| | 71.10 ($Pt^{0}$) | 38.7 | |
| Pt-SAs/$MoS_2$ | 73.50 ($Pt^{IV}$) | 8.2 | 1.74 |
| | 72.20 ($Pt^{II}$) | 70.6 | |
| | 71.50 ($Pt^{0}$) | 21.2 | |
| Pt-SAs/$MoSe_2$ | 73.78 ($Pt^{IV}$) | 6.7 | 2.13 |
| | 72.37 ($Pt^{II}$) | 93.3 | |
| Pt-SAs/$WSe_2$ | 73.95 ($Pt^{IV}$) | 30.2 | 2.60 |
| | 72.62 ($Pt^{II}$) | 69.8 | |

**Supplementary Table 6. Comparison of HER activity for Pt-SAs/TMDs in acidic solution with the state-of-the-art single-atom catalysts reported previously (electrolyte: 0.5 M $H_2SO_4$).**

| Catalyst | $\eta$ (mV) | Tafel slope (mV dec$^{-1}$) | $\eta_{10}$ (mV) | TOF (s$^{-1}$) | Reference |
|---|---|---|---|---|---|
| Pt-SAs/$WS_2$ | ~0 | 28 | 32 | 273@200 mV | This work |
| Pt-SAs/$MoSe_2$ | ~0 | 28 | 67 | 151@200 mV | This work |
| Pt-SAs/$MoS_2$ | ~0 | 31 | 59 | 172@200 mV | This work |
| Pt-SAs/$WSe_2$ | ~0 | 41 | 118 | 101@200 mV | This work |
| Pt@PCM | ~0 | 65.3 | 105 | 12@200 mV | *Sci. Adv.* 2018, 4, 6657 |
| A-Ni@DG | ~0 | 31 | 70 | 45@200 mV | *Chem* 2018, 4, 1 |
| $Pt_1$@Fe-N-C | ~0 | 42 | 60 | NA | *Adv. Energy Mater.* 2018, 8, 1701345 |
| Co-NG | ~30 | 82 | 147 | 1.19@200 mV | *Nat. Commun.* 2015, 6, 8668 |
| Ru SAs@PN | ~0 | 38 | 24 | 4.29@50 mV | *Angew. Chem. Int. Ed.* 2018, 57, 9495 |
| Pt/hCNC | ~0 | 24 | 15 | 7.67@20 mV | *Nat. Commun.* 2019, 10, 1657 |
| $Pt_1$/OLC | ~0 | 36 | 38 | 90@200 mV | *Nat. Energy* 2019, 4, 512 |
| W-SAC | ~0 | 58 | 105 | 4.5@120 mV | *Adv. Mater.* 2018, 30, 1800396 |
| $Pt_1$/NPC | ~0 | 28 | 25 | 100@100 mV | *ACS Catal.* 2018, 8, 8450 |
| $Ru_{SA}$-N-S-$Ti_3C_2T_x$ | ~0 | 90 | 76 | 1.50@200 mV | *Adv. Mater.* 2019, 1903841 |
| Pd/$MoS_2$ | ~0 | 80 | 89 | 16.54@200 mV | *Nat. Commun.*, 2018, 9, 2120 |
| $Mo_2TiC_2T_x$-$Pt_{SA}$ | ~0 | 30 | 30 | NA | *Nat. Catal.* 2018, 1, 985 |
| Co-NG-MW | ~0 | 80 | 175 | 0.385@100 mV | *Adv. Mater.* 2018, 30, 1802146 |
| Pt/NGNs | ~0 | 29 | ~40 | NA | *Nat. Commun.* 2016, 7, 13638 |

| | | | | | |
|---|---|---|---|---|---|
| Ni/graphene | ~50 | 45 | ~180 | 0.8@300 mV | *Angew. Chem. Int. Ed.* 2015, 54, 14031 |

Note that the red rows represent the acidic HER activity of Pt-SAs/TMDs in this study.

**Supplementary Table 7. Comparison of HER activity for Pt-SAs/$WS_2$ in acidic solution with the state-of-the-art Pt-based catalysts reported previously.**

| Catalyst | Loading ($\mu g_{Pt}\ cm_{disk}^{-2}$) | Electrolyte | Current density (mA $cm^{-2}$) | $\eta_{10}$ (mV) | Tafel slope (mV $dec^{-1}$) | Reference |
|---|---|---|---|---|---|---|
| Pt-SAs/$WS_2$ | 0.415 | 0.5 M $H_2SO_4$ | 54@100 mV | 32 | 28 | This work |
| PtW NPs/C | 20.38 | 0.5 M $H_2SO_4$ | NA | 19.4 | 27.8 | J. Am. Chem. Soc. 2020, 142, 17250 |
| PtFeCo | 51 | 0.5 M $H_2SO_4$ | 1325@400 mV | NA | 21 | Adv. Mater. 2016, 28, 2077 |
| Pt/$TiO_2$ | 0.21 ($\mu g_{Pt}$) | 0.5 M $H_2SO_4$ | 7@100 mV | 121 | 40 | Energ. Environ. Sci. 2017, 10, 2450 |
| CuPdPt/C | 0.27 | 0.5 M $H_2SO_4$ | ~120@100 mV | 55 | 25 | J. Mater. Chem. A, 2016, 4, 15309 |
| β-$Ni_2P_2O_7$/Pt | 1 | 0.5 M $H_2SO_4$ | 30@50 mV | 28 | 32 | ACS Appl. Mater. Interfaces 2019, 11, 4969 |
| AuPt NDs | 47.5 | 0.5 M $H_2SO_4$ | ~34@70 mV | 50 | 34 | Int. J . Hydrog. Energy 2016, 42, 18193. |
| Pt/def-$WO_3$@CFC | 15.9 | 0.5 M $H_2SO_4$ | ~28@100 mV | 42 | 61 | J. Mater. Chem. A, 2019, 7, 6285 |
| $Pt_{53}Ru_{39}Ni_8$ | NA | 0.5 M $H_2SO_4$ | NA | 37 | 34 | J. Colloid Interface Sci. 2017, 505, 14 |
| PtAg NCs | 59.8 | 0.5 M $H_2SO_4$ | 100@150 mV | ~60 | 40 | J. Colloid Interface Sci. 2017, 494, 15 |
| Pt NP/N-graphene | 5.65 | 0.5 M $H_2SO_4$ | 24@50 mV | 30 | 28 | Nanoscale, 2017, 9, 10138 |
| $Au_{38.4}$@$Au_{9.3}Pt_{52.3}$-NP/C | 17.16 | 0.5 M $H_2SO_4$ | 49.1@40 mV | 16 | 14 | Nanoscale, 2020, 12, 20456 |

| | | | | | | |
|---|---|---|---|---|---|---|
| 10Pt@HN-BC | 12 | 0.5 M $H_2SO_4$ | ~30@60 mV | 47 | 35 | Int. J. Hydrogen Energy 2018, 43, 6167 |
| H-PtNiCu-AAT | 26.5 | 0.1 M $HClO_4$ | ~70@100 mV | 32 | 33 | ACS Appl. Mater. Interfaces 2020, 12, 9600 |
| PtCoFe@CN | 13 | 0.5 M $H_2SO_4$ | ～16@50 mV | 45 | 32 | ACS. Appl. Mater. Interface 2017, 9, 3596 |
| $Pt_{75}Mo_{25}$/rGO | 76 | 0.5 M $H_2SO_4$ | ~65@100 mV | 32 | 32 | Carbon 2019, 146, 116 |
| PtPd@NSL | 19.7 | 0.5 M $H_2SO_4$ | ~55@50 mV | 29 | 23 | J. Electroanal. Chem. 2018, 822, 10 |
| 80Pt/C-MOF | 24.4 | 0.5 M $H_2SO_4$ | 50@50 mV | 42.1 | 24.45 | J. Mater. Chem. A 2019, 7, 20239 |

Note that the red rows represent the acidic HER activity of Pt-SAs/$WS_2$ in this study.

**Supplementary Table 8. Comparison between the previously reported Pt-doped $MoS_2$ and the single-atom Pt adsorbed atop $MoS_2$ for electrocatalytic HER.**

| Catalyst | Structure | Scheme | Active site | $\Delta G_H$ | Reference |
|---|---|---|---|---|---|
| Pt-SAs/$MoS_2$ | Pt adsorbed atop $MoS_2$ | 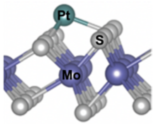 | Pt atoms | 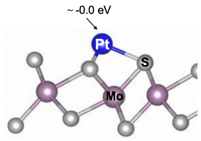 | This work |
| Pt-doped $MoS_2$ | Single-atom Pt doping (Mo substitution) | 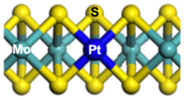 | S atoms | 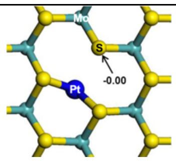 | Energy Environ. Sci., 2015, 8, 1594 |

**Supplementary References**


1 Deng, J. *et al.* Triggering the electrocatalytic hydrogen evolution activity of the inert two-dimensional $MoS_2$ surface via single-atom metal doping. *Energ. Environ. Sci.* **8**, 1594–1601 (2015).

2 Voiry, D. *et al.* Conducting $MoS_2$ nanosheets as catalysts for hydrogen evolution reaction. *Nano Lett.* **13**, 6222–6227 (2013).

3 Tang, Q. *et al.* Mechanism of hydrogen evolution reaction on 1T-$MoS_2$ from first principles. *ACS Catal.* **6**, 4953–4961 (2016).

4 Voiry, D. *et al.* Enhanced catalytic activity in strained chemically exfoliated $WS_2$ nanosheets for hydrogen evolution. *Nat. Mater.* **12**, 850–855 (2013).

5 Voiry, D. *et al.* Covalent functionalization of monolayered transition metal dichalcogenides by phase engineering. *Nat. Chem.* **7**, 45–49 (2015).

6 Lukowski, M. A. *et al.* Enhanced hydrogen evolution catalysis from chemically exfoliated metallic $MoS_2$ nanosheets. *J. Am. Chem. Soc.* **135**, 10274–10277 (2013).

7 Guo, X. *et al.* Few-layered trigonal $WS_2$ nanosheet-coated graphite foam as an efficient free-standing electrode for a hydrogen evolution reaction. *ACS Appl. Mater. Inter.* **9**, 30591–30598 (2017).

8 He, H. Y. Assembly of 1T-$WSe_2$: Sn nanosheets/graphene by a modified hydrothermal process for water splitting. *J. Sol-Gel Sci. Technol.* **93**, 554–562 (2020).

9 Zou, X. *et al.* Noble metal-free hydrogen evolution catalysts for water splitting. *Chem. Soc. Rev.* **44**, 5148–5180 (2015).
